# Altered mRNA Splicing in SMN-Depleted Motor Neuron-Like Cells

**DOI:** 10.1371/journal.pone.0163954

**Published:** 2016-10-13

**Authors:** Sara K. Custer, Timra D. Gilson, Hongxia Li, A. Gary Todd, Jacob W. Astroski, Hai Lin, Yunlong Liu, Elliot J. Androphy

**Affiliations:** 1 Department of Dermatology, Indiana University School of Medicine, Indianapolis, IN, United States of America; 2 Department of Medical and Molecular Genetics, Indiana University School of Medicine, Indianapolis, IN, United States of America; Iowa State University, UNITED STATES

## Abstract

Spinal muscular atrophy (SMA) is an intractable neurodegenerative disease afflicting 1 in 6–10,000 live births. One of the key functions of the SMN protein is regulation of spliceosome assembly. Reduced levels of the SMN protein that are observed in SMA have been shown to result in aberrant mRNA splicing. SMN-dependent mis-spliced transcripts in motor neurons may cause stresses that are particularly harmful and may serve as potential targets for the treatment of motor neuron disease or as biomarkers in the SMA patient population. We performed deep RNA sequencing using motor neuron-like NSC-34 cells to screen for SMN-dependent mRNA processing changes that occur following acute depletion of SMN. We identified SMN-dependent splicing changes, including an intron retention event that results in the production of a truncated Rit1 transcript. This intron-retained transcript is stable and is mis-spliced in spinal cord from symptomatic SMA mice. Constitutively active Rit1 ameliorated the neurite outgrowth defect in SMN depleted NSC-34 cells, while expression of the truncated protein product of the mis-spliced Rit1 transcript inhibited neurite extension. These results reveal new insights into the biological consequence of SMN-dependent splicing in motor neuron-like cells.

## Introduction

Spinal muscular atrophy (SMA) is a potentially fatal neurodegenerative disorder caused by the systemic depletion of the ubiquitously expressed survival motor neuron (SMN) protein [1]. While not entirely exclusive, motor neurons appear particularly vulnerable to the reduction of SMN, and emerging insights highlight the neuromuscular junction (NMJ) as a site or pre-pathological vulnerability [2]. Experimental evidence suggests that maintenance of the interface between nerve and muscle is particularly dependent upon the function of SMN within the nerve [3]. Despite advances in understanding the physiological pathology of SMA, the underlying mechanisms of motor neuron dysfunction resulting from SMN depletion remain elusive. Two prevailing hypotheses contend that SMN depletion results in aberrant mRNA splicing via a reduced capacity to assemble functional small nuclear ribonucleoproteins (snRNPs)[4], or defective mRNA localization to the peripheral neurite [5, 6].

The SMN protein is well documented to organize the assembly of RNA polymerase II derived small nuclear ribonucleic acids (snRNA) into a heptameric ring of Sm proteins followed by nuclear import of the mature snRNP [7–9]. SnRNPs form components of the spliceosome and disruption of these processes within motor neurons has been proposed to lead to alternative splicing of specific mRNAs essential to development and maturation of the neuromuscular junction [10].

An alternative hypothesis asserts that SMN is essential for transport and localization of mRNA into the neurite and presumably the growth cone [11–13]. *Ex vivo* neuronal cultures from SMA model mice show reduced presence of β-actin mRNA throughout the axon and growth cone as well as an inability to transport β-actin mRNA into the axon in response to extracellular cues [14, 15]. Attenuation of SMA pathology has been observed in a zebrafish model of SMA by over-expression of candidate plasticity-related gene 15 (cpg15), an mRNA known to be present in axons, which is found in complex with the neuronal RNA binding protein (RBP) HuD and SMN, suggesting that SMN-containing complexes are involved in translocation of mRNA species required for the health and maintenance of motor neurons [16]. Coupled with SMN active transport within the neurites of various culture models, SMN and the RNA binding protein hnRNP R have been visualized at the motor neuron synaptic terminal *in* vivo, demonstrating that this distribution pattern is not merely an artifact of neuronal culture [11].

Defining the individual RNAs that are aberrantly processed by either failure of the spliceosome or altered subcellular localization is technically demanding. Cell type-specific isoform expression patterns coupled with natural variation in splicing patterns during organismal development confound the identification of pathologically processed RNA transcripts *in vivo*. Likewise, processing and targeting of transcripts is likely to be equally diversified among the motor neurons, Schwann cells and muscle that comprise the neuromuscular architecture. The use of *in vitro* cultures provides the ability to accurately identify cell type specific alterations in RNA processing during controlled growth conditions. As Staropoli and colleagues pointed out, the majority of mRNA splicing changes within the spinal cord take place during normal development (postnatal day 1 vs. postnatal day 5), rather than between unaffected mice and SMA siblings at each age [17]. Cell-based systems remove this variable and allow for the study of the most basic facets of SMN biology. Prior investigations have been reported in this direction by the depletion of SMN in mouse fibroblasts and neuroblastoma cells [18, 19], however these cells do not possess the unique cytological architecture of the motor neuron, and thus important discrimination of subcellular compartments such as the developing neuronal processes is not represented in this analysis.

We chose to evaluate the role of the SMN protein in splicing using NSC-34 cells [20]. As we previously reported, these motor neuron-like mouse NSC-34 cells possess an SMA-like phenotype that can be restored to normal by expression of human SMN [21]. The advantage of this approach is the use of a uniform, clonal cell culture that is acutely and synchronously depleted of SMN protein. RNA-seq revealed transcriptome-wide abnormalities in splicing patterns following SMN depletion. Our analysis focused on differential splicing patterns and aberrant splicing events with intron retention. A number of novel truncated and elongated isoforms were identified following SMN depletion. Intron retention events were also evident following SMN depletion in a limited number of RNAs, most commonly resulting in the insertion of premature stop codons. Our analysis reveals the complexity of RNA processing events that are influenced by SMN levels in a cell type specific system and highlights the challenge of rescuing these events as a compensatory therapeutic strategy outside of specific restoration of SMN.

## Results

### SMN depletion reduces U snRNP biogenesis

**Fig 1:**
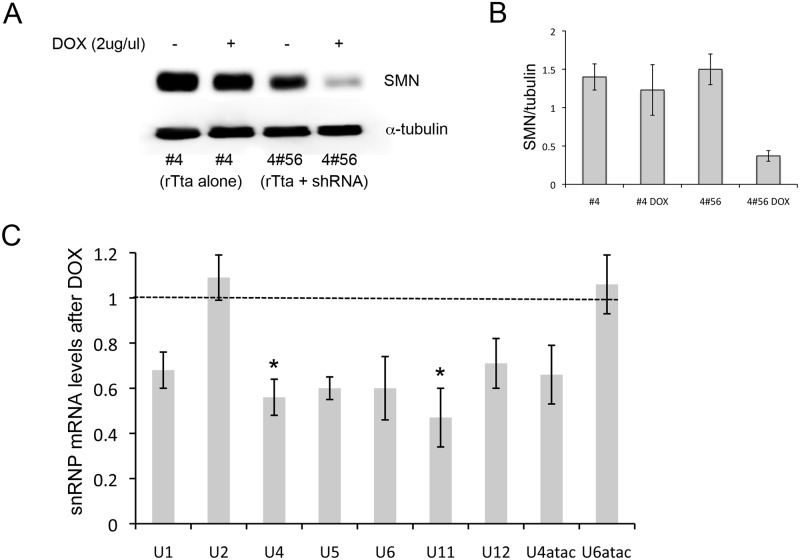
SMN depletion leads to defective U-snRNP biogenesis. A) Doxycycline treatment (2μg/ml) for 72 hrs reduced SMN protein levels by ~70% in NSC-34-4#56, which contain both the RTta and the SMN shRNA but not NSC-34 cell clone 4 which expresses only the RTta. B) Quantification of three separate western blot of SMN protein level after doxycycline treatment in NSC-34-4#56. C) Quantitative RT-PCR shows mRNA levels of UsnRNPs after doxycycline-induced SMN depletion compared to untreated cells (dashed line). All samples were compared to levels of the 18S subunit. *–*p*<0.01. One-way ANOVA followed by post-hoc t-test.

RNA for transcriptome analyses was isolated from NSC-34 clone 4#56 [21] using serum starvation to induce differentiation and neurite formation. Addition of doxycycline (2 μg/ml) for 72 hours induced expression of the SMN shRNA, which resulted in a ~70% reduction of the murine SMN protein as determined by Western blot. No decrease in SMN protein levels was seen following doxycycline treatment of NSC-34 clone #4 cells that express only the reverse Tet-transactivator (rTta) without the SMN shRNA (Fig 1a and 1b). As has been previously reported, SMN is involved in U snRNP biogenesis [22–24]. To determine if our NSC-34 system displays a similar phenotype, we harvested total RNA from control and SMN-depleted cultures followed by reverse transcription and quantitative PCR using previously described murine U snRNP primers [18, 24]. Consistent with the results seen in SMN-depleted NIH-3T3 cells, we detected significantly altered U snRNP mRNA levels (*p* = 0.0013 by one-way ANOVA), with post-hoc Tukey analysis revealing significantly decreased levels of U11 and U4 (*p*<0.01 denoted by asterisk, Fig 1c). These data suggest that spliceosome function could be altered in our NSC-34 cell model of SMA. There was a more significant decrease in U11 snRNA in SMN-depleted NSC-34 cells compared to SMN-depleted HeLa cells, which were reported to decrease U11 only when SMN levels had been reduced to 5% but not at 15% of control levels of SMN [8]. However, the overall profiles of snRNA changes in doxycycline treated Hela cells and our NSC-34 system are very similar after SMN depletion. A similar experiment with a lentiviral-expressed shRNA against SMN in undifferentiated MN1 cells found that only U11 was decreased, possibly due to the fact that this experiment was performed on a mixed population rather than a clonal cell line. Although our results showing decreased U11 and U4 agree with the findings in Hela cells, when snRNA levels were measured in multiple tissues from SMA mice, U11 was only significantly reduced in spinal cord and heart tissues from late stage mice (postnatal day 11) while U4 was only reduced in brain at postnatal day 6 [8].

### SMN depletion results in increased alternative splicing events

To address the question of alternative splicing events, we performed total transcriptome sequencing (RNA-seq). RNAs were harvested from three biological replicates of untreated or doxycycline-treated NSC-34-4#56 cultures and sequenced using the SOLiD platform. Following filtering and processing, 68–76% of all RNA sequence reads were mapped to the mouse reference genome assembly mm9 using an in-house pipeline that utilizes BFAST (0.7.0a). Unique reads with no more than two mismatches identified 12,168 genes representing the NSC-34 transcriptome. EdgeR was used to calculate differences in gene expression levels between untreated and doxycycline-treated samples by assuming the RNA-sequencing counts follow a negative binomial distribution. SMN shRNA-induced splicing changes were identified using software called Mixture of Isoforms (MISO) [25]. Based on a Bayesian inference framework, MISO is a probabilistic framework that quantitates the expression levels of alternatively spliced genes from RNA-seq data, and identifies differentially regulated exons across samples. MISO computes Percent Spliced In (PSI, or Ψ) values for each alternative splicing event, representing the fraction of a gene’s mRNA that includes the exon. For each event, MISO also calculates a Bayes Factor (BF) that quantifies the likelihood of the changes. For instance, BF = 5 indicates it is five times more likely that a specific alternative splicing event occurred than did not occur. Using BF of 5 as a cutoff, we identified a total of 145 events whose splicing patterns differ between control and SMN depleted cultures. Candidates were chosen for validation using end-point PCR by selecting targets with difference in PSI value (% of inclusion) greater than 0.5 between treatment groups and BF scores higher than 10 using samples obtained from fresh NSC-34-4#56 cultures with and without SMN shRNA induction as well as cells from clone #4 (rTta alone) with and without doxycycline. Altered mRNAs were subdivided into four groups: (1) those that predominantly undergo exon skipping producing a truncated splice variant with SMN depletion, (2) those that retain exons to predominantly express full length transcripts following SMN depletion, (3) those that retain introns following SMN depletion and (4) those that predominantly retain introns at normal SMN expression levels (Tables 1–4). Alternative exon splicing events predominated with relatively few (fifteen) alternative intron usage events. Table 5 details the total number of reads and the total percentage of mapped events. The complete dataset is available at http://compbio.iupui.edu/group/6/pages/smn and has also been submitted to to the NCBI sequence read archive (SRP090323).

**Table 1:** SMN-dependent exon skipping events ranked by percent inclusion.

Gene Name	Δ-psi	Baye's factor	Event
Tomm20l	-0.83	62.49	chr12:72218513:72218594:+@chr12:72222851:72222993:+@chr12:72224036:72224899:+
Ppp3cb	-0.73	10466467.31	chr14:21327846:21327943:-@chr14:21322469:21322498:-@chr14:21319252:21320884:-
Prmt1	-0.67	35020.18	chr7:52241653:52241938:-@chr7:52239450:52239503:-@chr7:52238856:52238957:-
Ppp2r3a	-0.65	1082.85	chr9:101027353:101027459:-@chr9:101019508:101019634:-@chr9:101007322:101010299:-
Rufy3	-0.6	7.69	chr5:89012085:89013342:+@chr5:89027461:89027514:+@chr5:89043966:89045604:+
Zbtb46	-0.6	7.21	chr2:181193961:181194132:-@chr2:181181407:181181589:-@chr2:181153313:181159093:-
Gyg	-0.59	5.2	chr3:20025144:20025363:-@chr3:20023008:20023058:-@chr3:20021967:20022750:-
Trmt5	-0.58	16869052884	chr12:74387671:74387976:-@chr12:74386891:74386962:-@chr12:74385198:74386262:-
Camkk2	-0.56	8.86	chr5:123187442:123187542:-@chr5:123186997:123187039:-@chr5:123181178:123184254:-
Pik3c2a	-0.55	10849865.75	chr7:123586818:123587034:-@chr7:123586105:123586192:-@chr7:123560967:123562095:-
Samhd1	-0.55	2935.02	chr2:156927457:156927594:-@chr2:156927057:156927179:-@chr2:156923265:156925239:-
Mettl9	-0.54	50.5	chr7:128200729:128200913:+@chr7:128216322:128216324:+@chr7:128219647:128219888:+
Slc44a2	-0.54	17.84	chr9:21156911:21156995:+@chr9:21157445:21157572:+@chr9:21158126:21159473:+
Fam35a	-0.52	8022922821	chr14:35122226:35122651:-@chr14:35082657:35082722:-@chr14:35079315:35082138:-
Nop58	-0.51	53505.63	chr1:59741784:59742070:+@chr1:59745414:59745463:+@chr1:59747330:59747833:+
Immt	-0.5	322.38	chr6:71802268:71802861:+@chr6:71803167:71803262:+@chr6:71806906:71807042:+
Mettl7a1	-0.5	11.45	chr15:100135192:100135775:+@chr15:100140255:100140335:+@chr15:100143425:100144797:+
Ube2l3	-0.5	6.9	chr16:17176505:17176600:-@chr16:17171856:17171954:-@chr16:17157390:17160269:-
Tmbim6	-0.49	303.79	chr15:99223310:99223471:+@chr15:99224126:99224161:+@chr15:99232011:99232094:+
Lrrc14	-0.48	7.69	chr15:76541068:76541266:+@chr15:76541394:76541513:+@chr15:76543297:76544415:+
Rap2c	-0.46	1460032.12	chrX:48368148:48368460:-@chrX:48361469:48361662:-@chrX:48357083:48359927:-
Nxt1	-0.46	8.36	chr2:148498337:148498571:+@chr2:148500200:148500334:+@chr2:148501018:148501771:+
Wdyhv1	-0.46	7.44	chr15:57982161:57982211:+@chr15:57985135:57985259:+@chr15:57989457:57990220:+
Myef2	-0.45	2743.52	chr2:124913413:124914747:-@chr2:124913279:124913325:-@chr2:124910364:124912452:-
Krit1	-0.45	22.35	chr5:3822087:3823785:+@chr5:3827628:3827761:+@chr5:3830606:3830772:+
Elk3	-0.44	197.37	chr10:92747514:92747722:-@chr10:92727625:92728425:-@chr10:92717434:92717556:-
Ptar1	-0.44	25.67	chr19:23783244:23783457:+@chr19:23792303:23792592:+@chr19:23794548:23795619:+
Gpatch2	-0.44	7.76	chr1:189049382:189050098:+@chr1:189053478:189053539:+@chr1:189054663:189054724:+
Maged1	-0.44	5.27	chrX:91787231:91787417:-@chrX:91786590:91786663:-@chrX:91785194:91785886:-
Dnajc5	-0.43	7.73	chr2:181283375:181283546:+@chr2:181283631:181283705:+@chr2:181283978:181289837:+
Znrf1	-0.42	6.47	chr8:114059994:114061464:+@chr8:114130647:114130748:+@chr8:114133193:114133288:+
Ywhab	-0.42	5.98	chr2:163837338:163837640:+@chr2:163837732:163837734:+@chr2:163839745:163839865:+
Rcor3	-0.41	8.64	chr1:193940150:193940227:-@chr1:193925633:193925748:-@chr1:193922425:193925036:-
Rhot1	-0.4	5.13	chr11:80068162:80068364:+@chr11:80071016:80071138:+@chr11:80079244:80081409:+
Hnrnpd	-0.38	324.99	chr5:100391534:100391631:-@chr5:100391117:100391223:-@chr5:100384954:100390352:-
Orai3	-0.38	93.11	chr7:134913328:134913730:+@chr7:134914248:134914392:+@chr7:134917071:134918668:+
Rps14	-0.37	2631505.51	chr18:60934164:60934340:+@chr18:60936055:60936205:+@chr18:60936584:60937062:+
Magi1	-0.37	11.97	chr6:93632946:93633084:-@chr6:93630783:93630871:-@chr6:93625447:93628996:-
Clstn1	-0.35	22.39	chr4:149012342:149012504:+@chr4:149015767:149015823:+@chr4:149017304:149017461:+
Stx11	-0.32	7.07	chr10:12683806:12684291:-@chr10:12663958:12664092:-@chr10:12659787:12661787:-
Josd2	-0.31	7.77	chr7:51723021:51723560:+@chr7:51723676:51723787:+@chr7:51724177:51725907:+
Aldoa	-0.29	49.79	chr7:133942559:133942769:-@chr7:133941488:133941492:-@chr7:133940851:133941290:-
Eif4h	-0.28	6.75	chr5:135101236:135101332:-@chr5:135100311:135100370:-@chr5:135097819:135097956:-
Pcmt1	-0.27	29.77	chr10:7368849:7368953:-@chr10:7368265:7368458:-@chr10:7367068:7367419:-
Hnrnpa2b1	-0.25	36.75	chr6:51419670:51420485:-@chr6:51417390:51417425:-@chr6:51417184:51417294:-
Hnrnpa2b1	-0.22	294.78	chr6:51416302:51416403:-@chr6:51414088:51414102:-@chr6:51411919:51413492:-
Stard4	-0.21	8.1	chr18:33365774:33365900:-@chr18:33364875:33364979:-@chr18:33358792:33363469:-

**Table 2:** SMN-dependent exon retention events ranked by percent inclusion.

Gene Name	Δ-psi	Baye's factor	Event
Cdc123	0.82	5.40E+273	chr2:5754702:5755114:-@chr2:5754127:5754159:-@chr2:5743067:5744243:-
Nrp2	0.72	1780.86	chr1:62832855:62832875:+@chr1:62859108:62859158:+@chr1:62861907:62865269:+
Fam64a	0.71	5.60E+71	chr11:71856424:71856839:+@chr11:71858483:71858760:+@chr11:71859181:71859595:+
Nubp1	0.7	10748107.36	chr16:10419746:10419778:+@chr16:10420349:10420439:+@chr16:10421067:10421530:+
Med7	0.67	20186.45	chr11:46250427:46250511:+@chr11:46253308:46253477:+@chr11:46254065:46256223:+
Phb2	0.64	8.85	chr6:124665972:124666048:+@chr6:124666443:124666448:+@chr6:124666624:124666967:+
Polh	0.61	1.28422E+34	chr17:46335583:46335717:-@chr17:46331151:46331365:-@chr17:46327556:46327725:-
Mtch2	0.61	8761.65	chr2:90689755:90689861:+@chr2:90692973:90693023:+@chr2:90693208:90693270:+
Afg3l1	0.6	178326.09	chr8:126022488:126022598:+@chr8:126023892:126024007:+@chr8:126025133:126025333:+
Ptrh2	0.6	102704.98	chr11:86497379:86497625:+@chr11:86501538:86501630:+@chr11:86503061:86505959:+
Serinc3	0.57	2.60E+109	chr2:163470734:163470889:-@chr2:163464902:163465063:-@chr2:163462543:163462740:-
Snrpd3	0.57	3.80E+74	chr10:74982059:74982205:+@chr10:74994922:74995114:+@chr10:74998006:75000126:+
Cse1l	0.57	88085852.77	chr2:166760253:166760389:+@chr2:166761423:166761526:+@chr2:166762842:166762939:+
Dcaf8	0.57	56.43	chr1:174078300:174079452:+@chr1:174095979:174096056:+@chr1:174102460:174104214:+
Stxbp1	0.56	6.29	chr2:32651536:32651690:-@chr2:32650108:32650233:-@chr2:32643123:32645157:-
Kars	0.55	4.9952E+12	chr8:114535110:114535255:-@chr8:114529532:114529688:-@chr8:114527175:114527340:-
Foxk2	0.55	9140760.75	chr11:121159822:121160169:+@chr11:121160898:121161107:+@chr11:121168082:121171214:+
Xpnpep3	0.55	159.21	chr15:81244862:81244978:+@chr15:81246277:81246424:+@chr15:81257706:81258384:+
Ptp4a2	0.54	6.67328E+34	chr4:129522306:129522436:+@chr4:129523709:129523783:+@chr4:129524958:129527231:+
Hnrnph1	0.54	4.73985E+15	chr11:50197182:50198238:+@chr11:50198643:50198692:+@chr11:50199279:50200031:+
Fam135a	0.53	370308.6	chr1:24039502:24039575:-@chr1:24037581:24037735:-@chr1:24035039:24037367:-
Neo1	0.52	494354.79	chr9:58736208:58736300:-@chr9:58732277:58732435:-@chr9:58728399:58728662:-
Pitpnc1	0.51	692.74	chr11:107087545:107087608:-@chr11:107077992:107078110:-@chr11:107069205:107073972:-
Mprip	0.5	2175532678	chr11:59585119:59585204:+@chr11:59585654:59585719:+@chr11:59589018:59595260:+
Cct4	0.5	17774127.29	chr11:22893267:22893319:+@chr11:22894298:22894387:+@chr11:22895930:22896038:+
Rbms3	0.5	26954.78	chr9:116495136:116495213:-@chr9:116491856:116491983:-@chr9:116481864:116487820:-
Isca2	0.5	95.1	chr12:86114256:86114627:+@chr12:86114743:86114858:+@chr12:86115493:86116043:+
Tarbp2	0.5	12.89	chr15:102348601:102349033:+@chr15:102349553:102349722:+@chr15:102351552:102354105:+
Tmem208	0.49	5.27	chr8:107852222:107852358:+@chr8:107852508:107852547:+@chr8:107852672:107852957:+
Crls1	0.47	20.87	chr2:132675602:132675739:+@chr2:132686945:132687030:+@chr2:132688071:132688139:+
Pank2	0.46	694.78	chr2:131099647:131099999:+@chr2:131105893:131106090:+@chr2:131108328:131108504:+
Ndufb5	0.45	6.40645E+17	chr3:32645331:32645436:+@chr3:32646519:32646711:+@chr3:32647383:32648279:+
Usp5	0.45	6.18	chr6:124767336:124767489:-@chr6:124767002:124767086:-@chr6:124765033:124765677:-
Kdm6a	0.44	16.56	chrX:17851637:17852287:+@chrX:17854502:17854689:+@chrX:17855752:17857062:+
Pstk	0.43	340.94	chr7:138514615:138514903:+@chr7:138517050:138517344:+@chr7:138517579:138517777:+
Wdfy2	0.43	5.39	chr14:63571849:63571979:+@chr14:63573698:63573806:+@chr14:63575117:63580346:+
Sltm	0.42	5	chr9:70390520:70390888:+@chr9:70391777:70391864:+@chr9:70406844:70410668:+
Nup88	0.41	9.38	chr11:70757614:70758240:-@chr11:70757381:70757499:-@chr11:70756560:70756824:-
Pxmp3	0.4	607616.71	chr3:5563165:5563272:-@chr3:5562669:5562732:-@chr3:5560498:5561764:-
Nup85	0.4	25117.84	chr11:115439244:115439378:+@chr11:115439478:115439600:+@chr11:115439803:115439934:+
Tyms	0.4	4431.92	chr5:30398174:30398247:-@chr5:30395006:30395078:-@chr5:30390386:30390726:-
Puf60	0.4	673.05	chr15:75905972:75906262:-@chr15:75905037:75905087:-@chr15:75902836:75902998:-
Shmt2	0.4	71.44	chr10:126957974:126958307:-@chr10:126957394:126957473:-@chr10:126957027:126957227:-
Prc1	0.4	11.96	chr7:87457918:87458017:+@chr7:87458413:87458454:+@chr7:87460000:87461202:+
Gpx8	0.39	7.37	chr13:113836380:113836621:-@chr13:113835640:113835901:-@chr13:113832691:113833507:-
Ube2v2	0.38	7109676.49	chr16:15581152:15581300:-@chr16:15577108:15577233:-@chr16:15551079:15556636:-
Uqcrh	0.38	4470.95	chr4:115747521:115747691:-@chr4:115743273:115743362:-@chr4:115742414:115742629:-
Polr3f	0.38	3402.14	chr2:144361911:144362126:+@chr2:144362902:144363093:+@chr2:144364348:144367731:+
Dnlz	0.38	11.2	chr2:26207293:26207631:-@chr2:26206855:26206994:-@chr2:26203075:26206078:-
Ptpmt1	0.38	5.68	chr2:90757586:90757969:-@chr2:90754141:90754332:-@chr2:90748507:90751476:-
Crls1	0.38	5.25	chr2:132675602:132675739:+@chr2:132680629:132680758:+@chr2:132686945:132687030:+
Ccnb1	0.37	10.87	chr13:101555412:101555573:-@chr13:101553420:101553602:-@chr13:101551580:101551738:-
Pisd-ps1	0.37	6.3	chr11:3025239:3026061:+@chr11:3028932:3029168:+@chr11:3029385:3029523:+
Bzw2	0.36	1846.79	chr12:36883255:36883451:-@chr12:36861478:36861542:-@chr12:36856579:36856755:-
Fxr1	0.36	87.52	chr3:33963025:33963241:+@chr3:33967083:33967174:+@chr3:33967839:33974920:+
Ppp2r2d	0.36	43.83	chr7:146060093:146060190:+@chr7:146061373:146061538:+@chr7:146062073:146062185:+
Syncrip	0.35	6.92	chr9:88376894:88377397:-@chr9:88375444:88375603:-@chr9:88374632:88374750:-
Tpm3	0.34	71	chr3:89891596:89894001:+@chr3:89894935:89895013:+@chr3:89903450:89904824:+
Phf3	0.33	5.89	chr1:30919832:30920101:-@chr1:30886688:30888404:-@chr1:30881055:30881364:-
Ppp1r12a	0.32	4923.07	chr10:107688797:107689000:+@chr10:107689813:107689980:+@chr10:107690381:107690551:+
Fundc1	0.31	557.02	chrX:17145159:17145234:-@chrX:17135807:17135935:-@chrX:17133690:17135137:-
Pcgf5	0.31	5.94	chr19:36517334:36517432:+@chr19:36519478:36519567:+@chr19:36530111:36535459:+
Tcp1	0.3	2.00129E+28	chr17:13109195:13109495:+@chr17:13110664:13110749:+@chr17:13110909:13113323:+
Ifi27l1	0.3	10290.22	chr12:104674773:104674892:+@chr12:104675663:104675734:+@chr12:104675886:104675915:+
Emd	0.3	34.72	chrX:71501082:71501215:+@chrX:71502217:71502263:+@chrX:71506121:71506935:+
Ddx17	0.3	29.97	chr15:79372917:79373016:-@chr15:79371464:79371597:-@chr15:79370839:79370908:-
Lypla1	0.3	12.73	chr1:4818665:4818730:+@chr1:4820349:4820396:+@chr1:4822392:4822462:+
Ndufs4	0.3	8.87	chr13:115178167:115178469:-@chr13:115141656:115141734:-@chr13:115107065:115107237:-
Srsf5	0.3	7.22	chr12:82047408:82047707:+@chr12:82048298:82048368:+@chr12:82048483:82048782:+
Cops3	0.29	38.46	chr11:59646386:59646435:-@chr11:59643649:59643741:-@chr11:59641348:59641527:-
Picalm	0.29	9.12	chr7:97326010:97326113:+@chr7:97330729:97330878:+@chr7:97337660:97337767:+
Serinc3	0.28	52.88	chr2:163470734:163470889:-@chr2:163464902:163464924:-@chr2:163462543:163462740:-
Mtch2	0.27	225498.8	chr2:90689755:90689861:+@chr2:90692997:90693023:+@chr2:90693208:90693270:+
Wbscr22	0.26	116.88	chr5:135532712:135532889:-@chr5:135531898:135532254:-@chr5:135529419:135529508:-
Ube2j2	0.25	19.92	chr4:155329531:155329669:+@chr4:155330477:155330557:+@chr4:155331190:155333713:+
Rai12	0.25	6.65	chr11:69784063:69784244:-@chr11:69782995:69783090:-@chr11:69782598:69782698:-
Sqle	0.24	12568724541	chr15:59155362:59155533:+@chr15:59156017:59156112:+@chr15:59157590:59157732:+
Agpat6	0.24	39.69	chr8:24301188:24302164:-@chr8:24295001:24295070:-@chr8:24293135:24293435:-
Surf4	0.24	6.16	chr2:26782289:26782475:-@chr2:26781128:26781204:-@chr2:26780473:26780516:-
Mrps18c	0.23	6.72	chr5:101230927:101231010:+@chr5:101232072:101232129:+@chr5:101232989:101235488:+
Znrf1	0.21	20.41	chr8:114059994:114061464:+@chr8:114133193:114133288:+@chr8:114143106:114143211:+
Slc30a9	0.21	6.2	chr5:67706917:67707084:+@chr5:67715913:67715972:+@chr5:67718092:67718191:+
Pot1a	0.2	149.81	chr6:25755875:25755941:-@chr6:25750284:25750510:-@chr6:25740138:25744674:-

**Table 3:** SMN-dependent intron skipping events ranked by percent inclusion.

Gene Name	Δ-psi	Baye's factor	Event
2500003M10Rik	0.41	227.54	chr3:90311467:90311548:-@chr3:90310030:90311180:-
Rpl7	0.26	317494357.1	chr1:16093684:16093765:-@chr1:16093591:16093650:-
Fasn	0.16	44.04	chr11:120671337:120671363:-@chr11:120671118:120671268:-
Nap1l1	0.11	7.64	chr10:110933096:110933161:+@chr10:110933810:110933939:+
Ptma	0.06	1.38696E+14	chr1:88426540:88426688:+@chr1:88426804:88426811:+

**Table 4:** SMN-dependent intron retention events ranked by percent inclusion.

Gene Name	Δ-psi	Baye's factor	Event
Vps33b	-0.55	242.16	chr7:87436001:87436176:+@chr7:87436221:87436465:+
Ing4	-0.5	29.19	chr6:124997500:124997958:+@chr6:124998194:124998255:+
Ibtk	-0.46	5.49	chr9:85611158:85611259:-@chr9:85608906:85609023:-
Rit1	-0.37	21035.04	chr3:88529917:88529990:+@chr3:88530202:88530393:+
Srsf1	-0.27	2668051.53	chr11:87862545:87862717:+@chr11:87862914:87867259:+
Cenpt	-0.25	123.29	chr8:108373733:108373823:-@chr8:108373526:108373613:-
Atg9a	-0.19	5.19	chr1:75179166:75179311:-@chr1:75177439:75178505:-
Ptbp1	-0.15	11.61	chr10:79322097:79322267:+@chr10:79322347:79322457:+
Psmb6	-0.07	9.27	chr11:70339387:70339454:+@chr11:70339796:70339927:+

**Table 5:** RNA-seq workflow and quality control.

Condition	total RNA control			total RNA SMN knockdown		
Sample	experiment 1	experiment 2	experiment 3	experiment 1	experiment 2	experiment 3
Total reads	30,113,572	30,088,415	26,659,894	29,684,299	39,127,193	33,014,903
Passed QC	25,254,611	23,761,419	22,115,822	24,304,285	32,199,505	25,683,819
Passed rRNA filter	13,265,153	19,533,367	20,479,028	1,384,802	9,273,930	10,518,125
(rRNA filter)	11,989,458	4,228,052	1,636,794	22,919,483	22,925,575	15,165,694
Mapped	10,043,543	13,298,811	15,517,242	1,033,316	6,849,638	7,303,336
Unmapped	3,221,610	6,234,556	4,961,786	351,486	2,424,292	3,214,789

**Fig 2:**
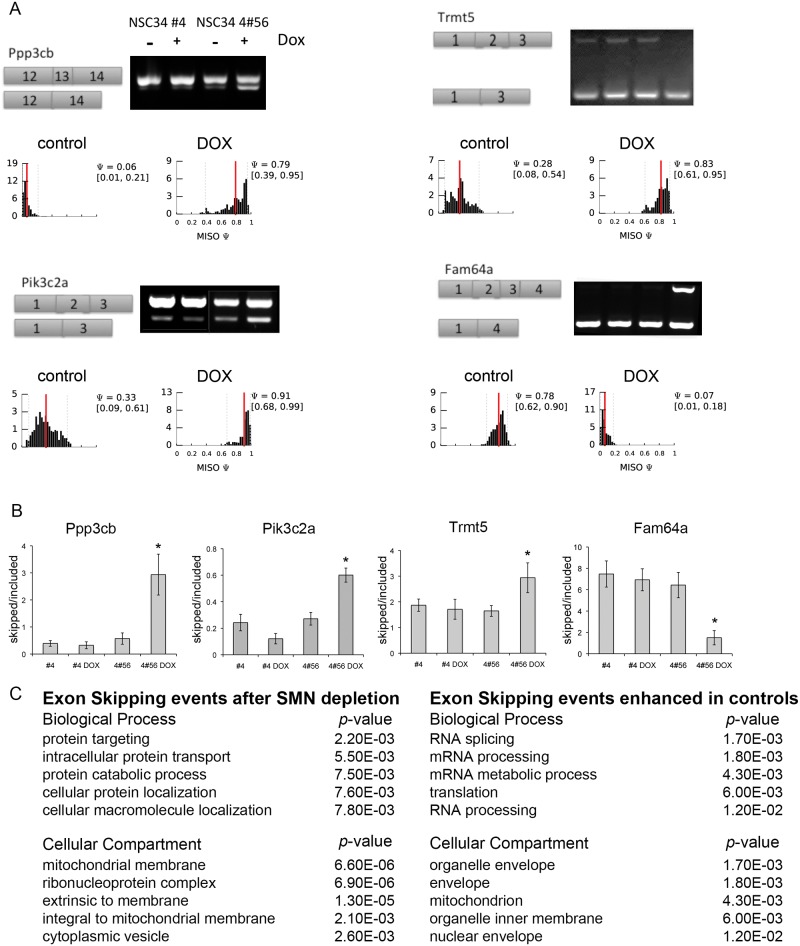
SMN depletion results in alternative exon splicing. A) RT-PCR documented alternative splicing events following SMN depletion for three group 1 and one group 2 transcripts. The histograms show the posterior distributions over PSI estimated by MISO in control or SMN-depleted samples conditions. The red lines depict the posterior mean and the dotted grey lines indicate 95% confidence intervals (also shown in parenthesis). B) Quantification of RT-PCR band intensity from 3 biological replicates showed increased alternative splicing after SMN depletion but not after doxycycline treatment of NSC-34-4. C) Gene Ontology results for exon skipping events and exon retention events induced by SMN depletion. *–*p*<0.01. One-way ANOVA followed by post-hoc t-test.

Three transcripts from group 1 that displayed exon skipping events following SMN depletion were selected for confirmation by RT-PCR analysis due to their high BF and PSI values: calcineurin A beta (*ppp3cb*), phosphoinositide-3-kinase class 2 alpha polypeptide (*pik3c2a*), and tRNA methyltransferase 5 homolog (*trmt5*). Primers were designed to the exons flanking the skipped exon, and using cDNA from NSC-34-4#56 as a template, the regions of interest were PCR amplified. All three genes exhibited increased levels of truncated transcript in SMN depleted samples (Fig 2a) but not in the control parental NSC-34 #4 cDNA. The histograms show the posterior distributions over PSI estimated by MISO [25, 26] in control or SMN-depleted samples conditions. The red lines show the posterior mean and the dotted grey lines indicate 95% confidence intervals.

The levels of exon-included versus exon-skipped transcripts were quantified from three separate experiments by band densitometry and each showed a significant impact of SMN depletion on the intensity of the alternatively spliced product (one-way ANOVA, Fig 2b). The fifty-two genes that produced truncated transcripts indicative of exon skipping following SMN depletion were queried using Gene Ontology analysis to identify underlying biological processes and cellular compartments that may be selectively vulnerable (Fig 2c). Skipped exon transcripts appear to function in subcellular protein trafficking and are components of mitochondrial membrane and vesicle complexes.

Family with sequence similarity 64 member A (*fam64a*) was chosen to validate group 2 transcripts with retained exons following SMN depletion, again because of its high BF and PSI values. As with skipped exons, clear differences were observed in the splicing of this target between control and SMN reduced NSC-34 cells while there was no difference observed in parental clone controls with doxycycline treatment (Fig 2a). 83 genes were identified that were predominantly transcribed as full-length in SMN-depleted cells, which gene ontology analysis indicated are involved in mRNA processing or localize to organelle membranes (Fig 2c).

To confirm that not all exons are affected by SMN depletion, we examined exon 19 on the RNA binding protein Fox1, which is expressed at high levels in NSC-34 cells and is known to be frequently alternatively spliced in neuronal cells [27]. Using published primers [28], we observed that exon 19 was skipped at equal levels in control cells treated with doxycycline or in #4–56 cells after doxycycline-induced SMN depletion (S1A1 Fig). To determine if alternative splicing impacted the overall expression level, quantitative RT-PCR was performed using primers against exons that are common to all splice variants. Although *Trmt5* and *Fam64a* expression levels were unchanged after SMN depletion, both *Ppp3cb* and Pik3c2a were significantly increased in doxycycline treated cells indicating that alternative splicing may result from an overall increase in expression (S1A2 Fig). All four validated alternatively spliced exons were examined by RT-PCR on cDNA isolated from SMA patient derived fibroblasts and healthy parent control (lines 3813T and 3814T respectively). End-point RT-PCR was unable to detect alternative splicing of either *Trmt5* or Fam64a. Exon 2 of *Pik3c2a* was skipped equally in SMA and parental fibroblasts. Only exon 13 of *Ppp3cb* was found to be preferentially alternatively spliced in the SMA cells (S1A3 Fig).

**Fig 3:**
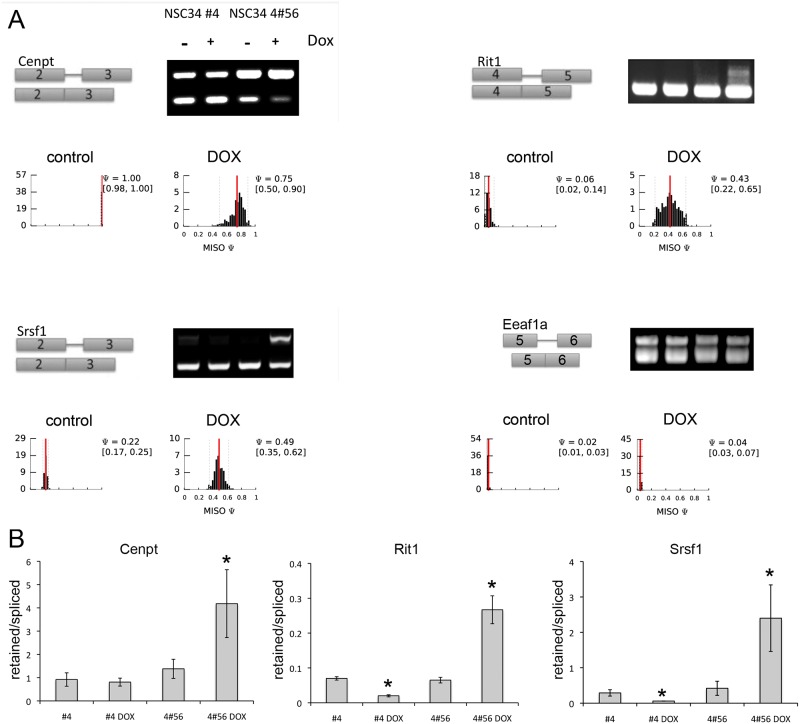
SMN depletion results in alternative intron usage. A) RT-PCR demonstrated increased intron retention events in doxycycline treated NSC-34-4#56, but not NSC-34-4. The histograms show the posterior distributions over PSI estimated by MISO in control or SMN-depleted samples conditions. The red lines depict the posterior mean and the dotted grey lines indicate 95% confidence intervals (also shown in parenthesis). B) Quantification of RT-PCR band intensity from 3 biological replicates shows increased intron retention after SMN depletion, but not after doxycycline treatment of NSC-34-4. *–*p*<0.01. One-way ANOVA followed by post-hoc t-test.

MISO was used to analyze intronic sequence retention from NSC-34 transcriptome reads comparing cells with wild type and depleted levels of SMN protein. Using a Bayes Factor cutoff value of 5, fourteen examples of alternatively spliced introns were observed in SMN-depleted cells compared to controls (Fig 3). Validation of intron retention was performed by selecting candidates with large differences in the percentage sequence inclusion (ΔPSI) and Bayes Factor scores greater than 5. Using these criteria, cDNA from NSC34-4#56 was screened using primers flanking centrosomal protein T (*cenpt*) intron 2, Ras-like without CAAX1 (*rit1*) intron 4, and serine/arginine-rich splicing factor 1 (*srsf1*) intron 3. All targets analyzed displayed intron retention following SMN depletion whereas analysis of the parental clonal line did not show any difference in intron retention following doxycycline treatment (Fig 3a). RT-PCR primers designed against the same introns in the human genes did not detect retention of these introns in cDNA from SMA 3813T fibroblasts compared to healthy parental 3814T fibroblasts (S1A4 Fig). The histograms show the posterior distributions over PSI estimated by MISO in control or SMN-depleted samples conditions. The red lines show the posterior mean and the dotted grey lines indicate 95% confidence intervals. Quantification by band densitometry of multiple experiments showed a significant increase in intron retention with SMN-depletion (*p*<0.01 by one-way ANOVA, Fig 3b). For comparison, MISO analysis predicted that Eef1a1 was expressed at high levels and that retention of intron 6 showed no significant difference between control and SMN-depleted samples. Primers anchored in the flanking exons show a strong RT-PCR product under all conditions that was unaffected by SMN depletion (Fig 3a), demonstrating that SMN-dependent intron retention events are specific to individual targets rather than a more widespread splicing error.

### U11/U12 splicing after SMN depletion

**Fig 4:**
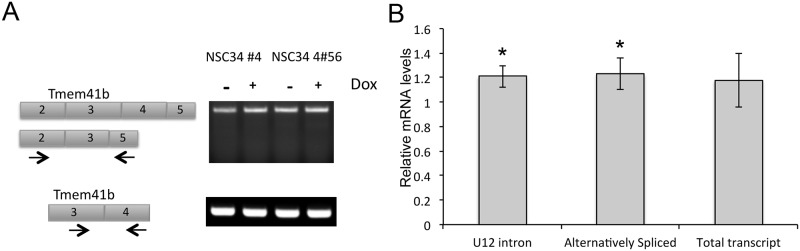
*Tmem41b* is alternatively spliced in SMN-depleted NSC-34 cells. A) Endpoint RT-PCR shows no alternative splicing of *Tmem41b* or retention of the U12 dependent intron 3 in either parental cell line NSC-34 #4 or the doxycycline-inducible SMN knockdown line NSC-34 #4–56 following 72 hours of doxycycline. B) Quantitative RT-PCR shows a significant 20% increase in U12 intron retention and alternative splicing in *Tmem41b* after doxycycline-induced SMN depletion but no decrease in total *Tmem41b* levels. *–*p*<0.05 by Student’s t-test.

Primary focus was placed on U12 introns during MISO analysis due to previous reports of altered non-canonical splicing errors following SMN knockdown [18]. However, only two U12 dependent introns were found to be affected. Introns 1 and 2 of Bzw2 were preferentially retained under control conditions (Δpsi = 0.36) and intron 8 of Myef2 was retained after SMN depletion (Δpsi -0.45). This analysis indicates that in NSC-34 cells, non-canonical splicing is unaffected by SMN depletion. While it has been reported that the *tmem41b* transcript is differentially spliced in SMN depleted NIH-3T3 fibroblasts [18], we were unable to detect the reported alternative splicing resulting from the exon3-5 fusion or the retention of intron 4 by endpoint RT-PCR (Fig 4a) using previously published primers anchored in exons 2 and 5 [18]. Our design of primers anchored in exons 3 and 5 yielded a similar result, detecting only the properly spliced product under all conditions (Fig 4a). Using the previously published qPCR primers [18], we were able to detect significant increases in the retention of the U12-dependent intron 3 as well as the alternatively spliced product after SMN depletion (Fig 4b), but found overall expression levels were very low. Using primers designed to detect all *tmem41b* isoforms [18], we detected a similar increase in total levels after SMN depletion. This is in contrast to the findings in NIH-3T3 cells in which alternative splicing resulted in an overall decrease in *tmem41b* transcript levels [18]. The previous report of altered *tmem41b* splicing was following SMN depletion for five days rather than the three-day knockdown used here. Our findings do, however, highlight the impact of using disease relevant cell types for the interrogation of SMN-dependent splice changes.

### Identification of RNA binding protein motifs in SMN-dependent exon skipping events

**Fig 5:**
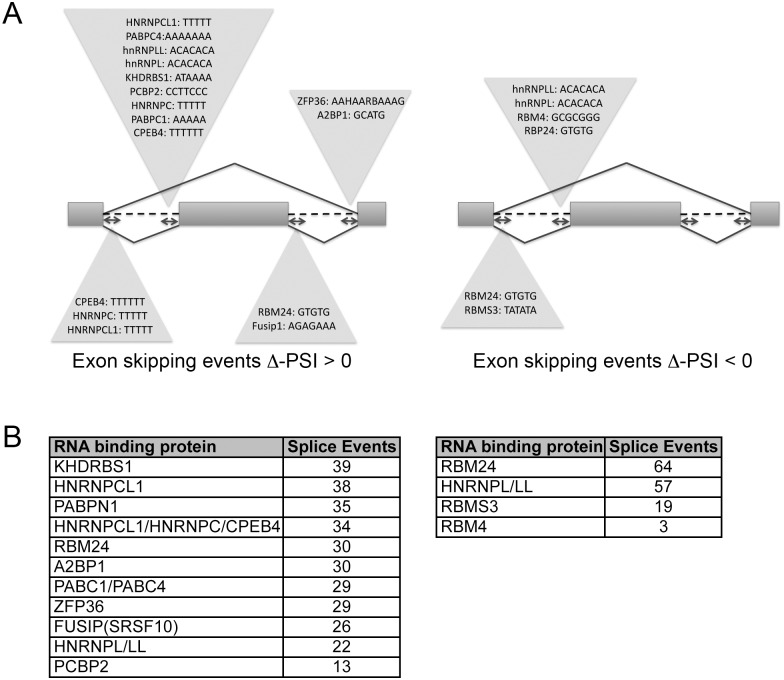
RNA binding protein motifs at sites of alternative exon splicing. A) The upstream and down-stream intronic regions for each alternatively spliced exon were interrogated for the presence of pentameric RNA binding protein motifs. No significant RBP recognition sites were identified within exons or in the up- or downstream exon. B) The total number of recognition motifs for each RNA binding protein is displayed. Only hnRNP-L/LL and RMP4 binding motifs were present at both retained and skipped exons.

SMN has been shown to interact with RNA binding proteins (RBPs) so we hypothesized that perhaps a common RBP was regulating SMN-dependent splice events in NSC-34 cells. In order to identify candidate RBPs, we analyzed the upstream and downstream exons, the entirety of the alternatively spliced exon, and the intervening introns using previously described methods [29] for pentameric motifs associated with known RNA binding proteins (Fig 5a). The candidates were screened using a strict cut off of Bayes factor greater than 10 and a false discovery rate (FDR) less than 0.05, we identified 76 splicing events with Δ-PSI greater than zero (increased inclusion levels) and 35 with Δ-PSI less than zero (decreased inclusion level). Thirteen RBPs were identified (Fig 5b), one of which is a well-characterized regulator of alternative splicing: FUSIP/SRSF10 [30]. Two of its validated splice targets, KDM6a and SLTM, are present in our list of alternatively spliced exon events following SMN depletion, indicating that perhaps in conditions of low SMN, FUSIP/SRSP10 occupancy at these regulatory regions was decreased. Only hnRNP-L/LL and RBM24 binding motifs were present at exons that are both included or skipped after SMN-depletion, indicating that these RNA binding proteins could be master co-regulators of SMN-dependent alternative splicing events. None of the RBPs predicted to regulate the alternatively spliced exons and introns were themselves mis-spliced after SMN depletion. Several RBPs themselves are alternatively spliced after SMN knockdown, but since none of them are predicted to bind the identified splice sites, it is unlikely that these mis-spliced RBPs are responsible for the observed alternatively spliced transcripts.

### Expression of SMN restores normal splicing patterns

**Fig 6:**
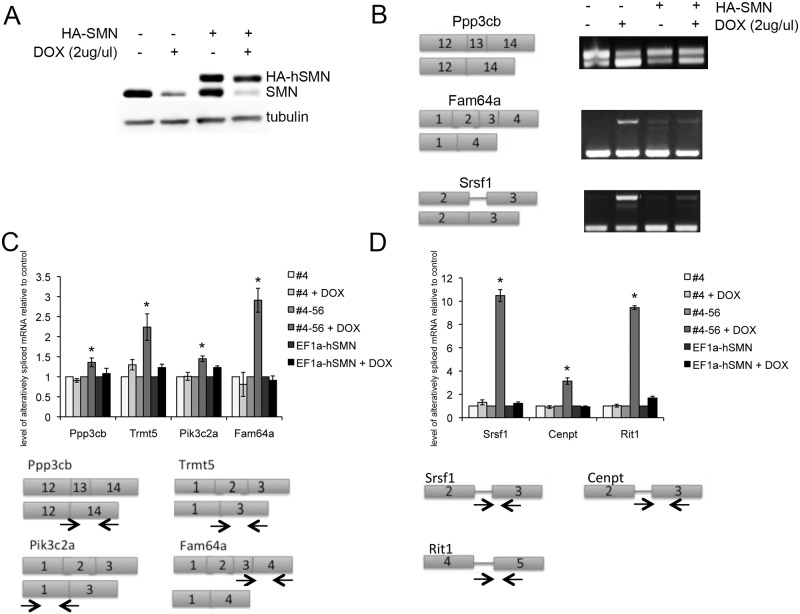
Aberrant splicing is rescued by reintroduction of human SMN. A) NSC-34-4#56 cells were transfected with HA-tagged human SMN. Western blotting with antibodies against SMN (MANSMA1) showed that doxycycline reduced the levels of mouse SMN but the HA-hSMN was unaffected and brought levels of SMN protein within normal range after 72 hours in doxycycline. B) RT-PCR on SMN-depleted and HA-hSMN expressing cultures shows rescue of three major splicing change categories. C-D) Quantitative real-time PCR (qRT-PCR) demonstrated that the aberrant splice products from exon skipping/retention (6C) or intron retention (6D) were only present after doxycycline-induced SMN depletion, and normalized with expression of human SMN. Schematic shows the location of primers for qRT-PCR quantification of alternative splice products.

To confirm that the alternative splicing events identified by RNA-seq and verified by RT-PCR are true SMN-dependent splicing changes, we used a human HA-tagged SMN cDNA that is not affected by the murine-specific shRNA in the 4#56 clone to restore SMN protein levels to normal. NSC-34 4#56 were transfected with HA-hSMN and pBabe-hygro, and a polyclonal population was used for validation experiments. Western blot analysis with antibodies against SMN showed expression of this HA-hSMN protein was stable in the presence of doxycycline without disrupting the knockdown of murine SMN (Fig 6a). RNA was harvested from these HA-hSMN rescued cultures and alternative splicing interrogated by RT-PCR. We selected representative candidates from three categories of splice error; exon skipping, exon retention and intron retention, and demonstrated that expression of SMN protein restored these splicing patterns to normal (Fig 6b). Finally, we measured the amount of each validated splice variants by quantitative real-time PCR on RNA isolated from NSC-34 line #4 (rTta alone), line #4–56 (SMN shRNA) and line #4–56 expressing human HA-SMN (EF1a-hSMN) with and without doxycycline normalized to 5S RNA by the ΔΔCt method and values from untreated cells were set to 1. For exon skipping products, one primer was placed across an exon-exon border. For intron retention events, one primer was anchored within the intron and the other in the flanking exon. The end-point PCR shown in Fig 6b shows aberrant splice products were present after SMN depletion in line #4–56 and that the profile normalized with the addition of human SMN for both exon skipping and retention (Fig 6c) and intron retention events (Fig 6d).

### Transcripts with retained introns are stable

**Fig 7:**
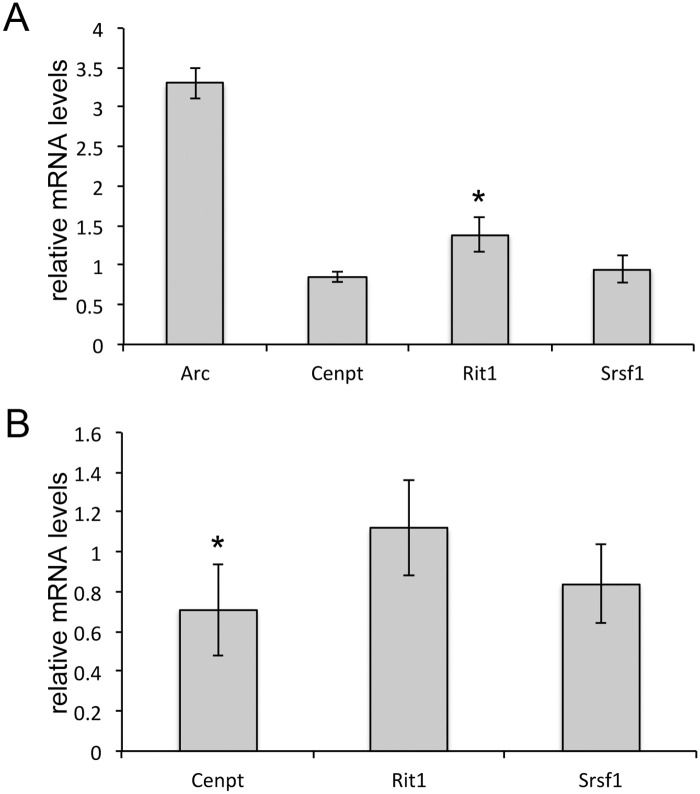
Retained introns are not targets of non-sense mediated decay. A) Quantitative RT-PCR measures intron-retained transcripts after treatment with cycloheximide. mRNA levels were compared to vehicle treated (EtOH) cultures using 5S rna as the internal reference by the ΔΔCT method. *Arc*, a validated NMD target increased while only *Rit1* showed any significant increase, indicating that these transcripts are not targets of NMD. B) Quantitative RT-PCR compared total transcript levels in SMN-depleted cultures to control cultures using 5S rna as the internal reference by the ΔΔCT method. *Cenpt* was slightly decreased after SMN depletion, but neither *Srsf1* nor *Rit1* transcript are decreased overall after SMN depletion. (Asterisk–*p*<0.05 by Student’s t-test).

For all three of the group 3 validated intron retention events, the resultant transcript contains a premature stop codon, which we might direct it to the nonsense mediated decay pathway (NMD). However, recent studies in neuronal cells and mouse cerebellum have demonstrated that intron retention can be an important tool for expanding the available transcriptome in neuronal cells, and that intron retained transcripts can be stable and produce truncated protein products [31]. To determine if the transcripts with SMN-dependent intron retention are targets of NMD, we followed published methods and exposed the SMN-depleted cultures to the protein synthesis inhibitor cycloheximide (10 μM) for 4 hours [32]. As a positive control, we used the well-characterized NMD target *Arc* [33], which should increase after inhibition of the NMD machinery proteins by cycloheximide. Quantitative RT-PCR with primers specific to the intron-retained transcripts shows that their levels are relatively unchanged after cycloheximide treatment whereas *Arc* transcript increased more than 3 fold (Fig 7a). Intron retention could be used to regulate the over all transcript levels, so we designed primers to common exons and found that only *Cenpt* decreased after SMN-depletion. Total transcript levels for *Rit1* and *Srsf1I* were not significantly changed (Fig 7b).

### SMN-dependent splice errors are increased in SMA model mice

**Fig 8:**
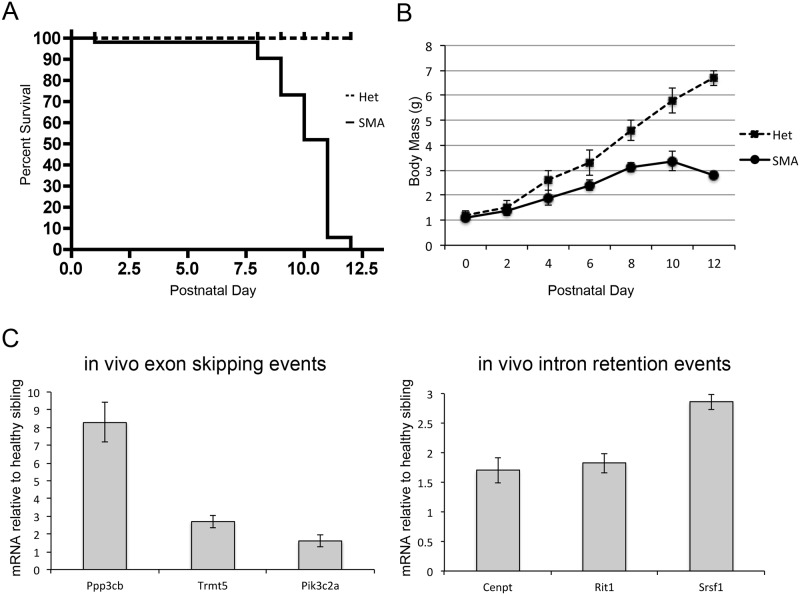
Alternative splicing in a mouse model of SMA. A) Kaplan-Meier analysis shows a significantly decreased lifespan in SMA mice compared to heterozygous (Het) littermates (*p<0*.*01 n* = 50). B) SMA mice fail to gain and maintain weight compared to the Het siblings (*p*<0.01 by two-way ANOVA). C) At postnatal day 9, SMA spinal cords show increased levels of exon-skipped transcripts and intron-retained transcripts compared to Het spinal cords (p<0.05 by Student’s t-test).

To determine if the SMN-dependent alternative splice products in our NSC-34 cell model were present in a mouse model of SMA, we chose the so called “Taiwanese” model [34]. The mice were obtained from Jackson Labs (stock # 005058) and crossed with FVB/NJ females as described by Gogliotti et al [35] to produce litters that are 50% healthy heterozygotes and 50% SMA. In our experience, these mice live approximately 12 days. Kaplan-Meier analysis shows a significant decrease in survival compared to healthy siblings as well as a failure to maintain body mass (Fig 8a and 8b). Lumbar spinal cord was harvested at postnatal day 9, when the SMA pups are fully symptomatic as evidenced by significant weight loss but are still ambulatory. Quantitative RT-PCR was run using primers specific for the validated alternatively spliced transcripts. All three of the group 1 exon-skipping events were significantly increased in SMA mice. The three validated intron-retention events were also significantly increased compared to healthy siblings. (Fig 8c).

### Biological Consequences of splicing errors in SMN-depleted cells

**Fig 9:**
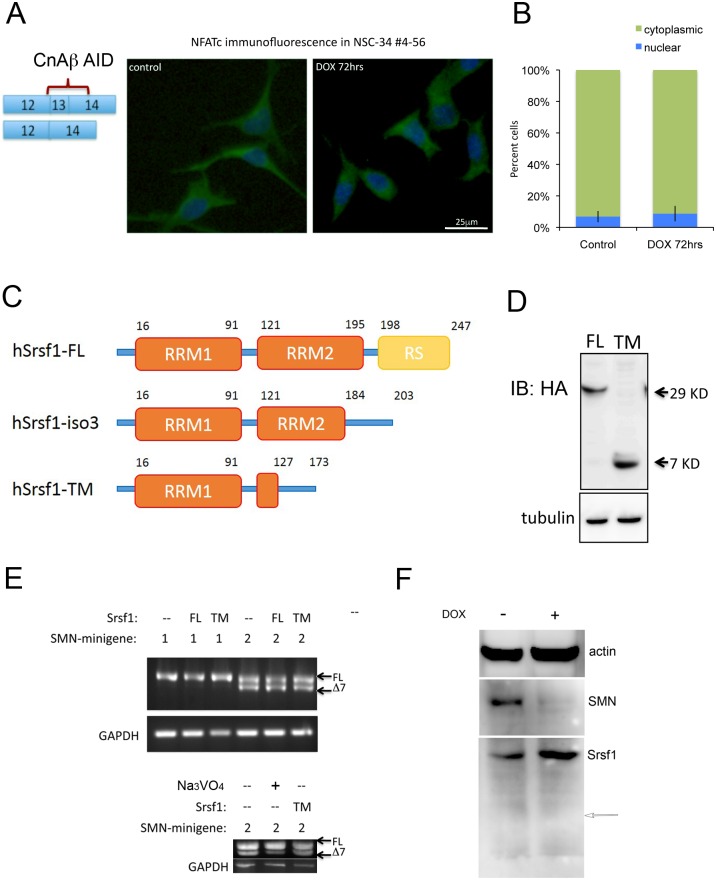
Alternatively spliced CnAβ and Srsf1 do not impact normal biology. A) Immunofluorescent detection of NFATc localization (green) in NSC-34 4#56 cultures showed that SMN knockdown did not increase nuclear localization of NFATc. Nuclei were visualized with DAPI (blue). B) Quantification of NFATc localization in three separate experiments showed no significant effect of SMN depletion. C) Schematic of Srsf1 protein domains. RRM: RNA Recognition Motif, RS: Arginine/Serine rich region, FL: full length, Iso3: naturally alternatively spliced dominant negative product, TM: alternatively spliced truncation mutant induced upon SMN knockdown. D) FL and TM Srsf1 expression. FLAG Western blot of FLAG-Srsf1 constructs expressed in NSC-34-4#56 cells. E) Srsf1 expression did not affect SMN splicing. RNA was harvested from NSC-34-4#56 cells transfected with FLAG-Srsf1 FL/TM and SMN1/2 minigene plasmids. *SMN1* and *SMN2* were PCR amplified and run on an agarose gel. Addition of 100μM Na_3_VO_4_ shows that the SMN2 minigene is functional, increasing the amount of exon 7 included transcript. F) Endogenous Srsf1-TM was not detectable upon SMN knockdown. Western blotting with N-terminal Srsf1 antibody of control and DOX treated NSC-34-4#56 cells detected the full length Srsf1 protein, but not the predicted Srsf1-TM (arrow).

The validated splicing change in *Ppp3cb* affects the protein calcineurin Aβ(CnAβ), a neuron-specific phosphatase. This splice error causes exclusion of exon 13, which results in loss of a small fragment of the protein’s auto-inhibitory domain (AID) [36]. We predicted that this change would result in increased CnAβ activity. To explore this hypothesis, we used immunofluorescence to study the intracellular localization of the transcription factor NFATc. Phosphorylated NFATc is retained in the cytoplasm but following dephosphorylation by CnAβ, translocates into the nucleus [37]. Hyperactive CnAβ would result in increased NFATc nuclear immunofluorescence. NSC-34 cells from clone 4#56 were grown in the presence or absence of doxycycline for 72 hrs and immunofluorescently labeled with antibodies against total NFATc. Cultures were grown in calcium-free media to reduce endogenous CnAβ activity. Under these conditions, NFATc is exclusively cytoplasmic and doxycycline-induced SMN depletion produced no visible increase in nuclear staining (Fig 9a). Quantification of the localization of NFATc immunofluorescence from three separate experiments demonstrated no significant change in the percentage of cells with nuclear NFATc staining after SMN depletion (Fig 9b). It remains possible that levels of truncated calcineurin are simply too low to have a biological effect, or the region of the auto-inhibitory domain that is removed is too small to impact normal protein function.

Having determined that our validated intron-retained transcripts were not targets of nonsense mediated degradation, we designed a series of experiment to determine the consequences of translation into a truncated protein product. Neuronal cells are capable of translating intron retained transcripts in a developmentally regulated fashion, and this process can be extremely important for local translation in the axon. For instance, Robo3.2, an intron-retained isoform, is actively translated into protein in cultured commissural axons [38]. Retention of the intron between exons 2 and 3 in *Srsf1* was very prominent and appeared to be completely negated following expression of HA-tagged human SMN (Fig 4). Inclusion of this intron creates an in-frame stop codon that truncates the arginine-serine rich domain (RS) and most of the RNA recognition motif (RRM2) in the C-terminal 128 amino acids of Srsf1 (Fig 9c). Several natural isoforms of *Srsf1* exist including isoform 3, which similarly lacks RS and part of RRM2 (Fig 9c) and was previously found to be dominant negative [39]. In view of this, we sought to determine if the SMN dependent truncation product similarly exhibited an effect on splicing. HA-tagged full-length (FL) and truncated (TM) human Srsf1 were cloned for use in an *in-vitro* splicing assay where the splicing template is exon 7 *SMN1* or *SMN2* [40]. Srsf1 binds the exonic splicing enhancer (ESE) to promote inclusion of exon 7 in *SMN1*, while *SMN2* has a variant in the ESE sequence (C840T) that blocks Srsf1 binding and results in exon skipping [41]. NSC-34-4#56 cells were transfected with either hSrsf1-FL or TM along with the SMN reporter. Three days later, RNA was harvested, PCR amplified, and run out on an agarose gel. Cells transfected with only the *SMN1* reporter have a single higher band corresponding to SMN with exon 7 included, while the *SMN2* reporter produced an additional band corresponding to the Δ7 transcript (Fig 9e). Co-transfection of hSrsf1-FL or TM in this system successfully produced stable protein (Fig 9d), however, no change in splicing ratios was detected in either *SMN1* or *SMN2* mini-cassettes (Fig 9e), suggesting this Srsf1 truncation product was not dominant negative and may have no effect on splicing of SMN. As a positive control, NSC-34 cells transfected with the SMN2 minigene were treated with 100μM Na_3_VO_4_ overnight, which has previously been shown to promote correct splicing and increase exon 7 inclusion [42]. As expected, Na_3_VO_4_ increased the level of full-length transcript, while hSrsf1-TM did not (Fig 9e). This non-functional mutant is in the context of human Srsf1 and not the more relevant mouse Srsf1. The mSrsf1 truncation mutant similarly lacks the RS domain and most of RRM2, but when HA-mSrsf1-TM was cloned and expressed in NSC-34 cells, a stable protein was not detected by western blot analysis. Probing endogenous murine Srsf1 protein by Western blot in NSC-34 cells using an N-terminal antibody did not reveal any truncated protein products upon SMN knockdown (Fig 9f, arrow), further strengthening the observation that mSrsf1-TM does not produce a stable protein. The Western blot in Fig 9f does demonstrate clearly that Srsf1 protein levels are not reduced by SMN-depletion, indicating that the intron-retention event is not leading to changes in the overall levels of full-length Srsf1 protein. In light of the lack of mSrsf1-TM expression and the absence of any splicing effect of hSrsf1-TM, the splicing change in *Srsf1* upon SMN knockdown is unlikely to contribute to the SMA phenotype.

**Fig 10:**
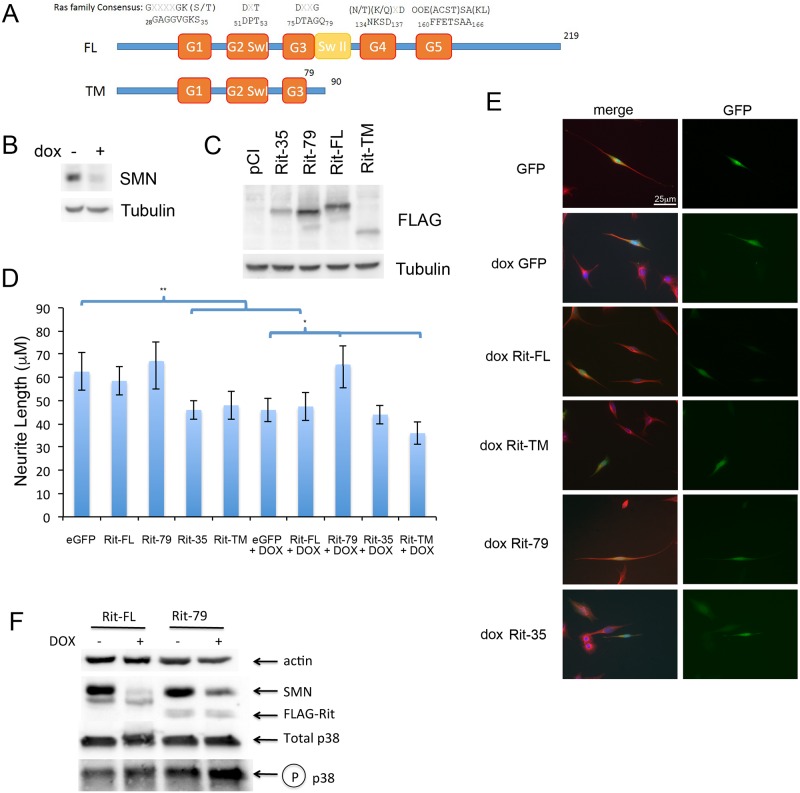
Alternatively spliced Rit1 decreases neurite length. A) Schematic of Rit1 protein domains. Protein sequence shown of minimal consensus G1-G5 domains of Ras family members on top followed by the mouse Rit1 specific sequence and corresponding amino acid position. SwI: Switch I, SwII: Switch II domains, FL: Full length, TM: truncation mutant. B) Representative levels of SMN for NSC34-4#56 cells used in panels D and E upon dox-induced knockdown. C) Western blot expression of FLAG tagged dominant negative Rit S35N, constitutively active Rit Q79L, full length Rit (FL), and alternatively spliced Rit truncation mutant (TM). D) Neurite extension assay. NSC34-4#56 cells were dox treated for 3 days followed by transfection of FLAG-Rit /GFP for 3 days. Cells were fixed and tubulin stained to allow neurite length measurement in GFP positive cells. Data are representative of three independent experiments, p<0.001 as determined by one-way ANOVA and post-hoc Bonferroni test. **: p<0.01. E) Immunofluorescent images of representative cells. Red: Tubulin, Green: GFP, Blue: DAPI nucleus. F) Western blot from NSC-34 cells following differentiation and 72 hours treatment with doxycycline. Transfection with Rit-79 but not Rit-FL increased levels of phospho-p38 and SMN in doxycycline-treated cultures.

Rit1 is a small G protein involved in promoting axonal outgrowth [43]. We chose this intron retention event because the dominant phenotype seen in NSC-34-4#56 cells is a reduced neurite length [21, 44], and Rit1 has been shown to influence neurite development in cultured neurons [45]. Upon SMN depletion, Rit1 intron 4 is retained resulting in the inclusion of a stop codon that prevents transcription of the switch II, G4, and G5 domains [46] normally required for proper orientation of GTP as well as downstream effector proteins (Fig 10a) [47]. If these missing domains render the truncation mutant (Rit-TM) functionally inactive, we would expect that expression of Rit-TM in NSC-34 cells would not promote neurite outgrowth. To examine this, NSC-34-4#56 cells were treated with doxycycline for 3 days to reduce SMN levels (Fig 10b) and thereby reduce neurite length before being transfected with GFP as a marker and FLAG-tagged Rit-FL, Rit-TM, constitutively active Rit-Q79L, or the dominant negative Rit-S35N mutant [48]. Three days after transfection, cells were either lysed and used for Western blot analysis, or fixed for staining with DAPI and tubulin to measure neurite lengths. Under this protocol, neurites are predominantly Map2 positive dendrite-like structures, and only processes more than twice the width of the soma are considered a neurite (S2A1 Fig). After 72 hours of differentiation, RT-PCR shows that cultures are expressing neuronal markers such as ChAT, Map2 and Tau, and greater than 70% of the cells produce at least one neurite (S2A2 and S2A3 Fig). All four Rit constructs produced stable proteins as detected by Western blot (Fig 10c). The slightly larger size of Rit-FL compared to Rit-79 or -35 can be attributed to Rit-FL’s 4x FLAG-tag while the latter have a 3x FLAG. Reduced expression of Rit-S35N but not Rit-Q79L was previously documented and was observed here [49]. Constitutively active Rit-79, which cannot hydrolyze GTP and therefore continuously activates downstream effectors [48], restored neurite length in SMN-depleted cultures. The dominant negative Rit-S35N reduced neurite length even more than SMN-depletion, as summarized in Fig 10d. Interestingly, Rit-TM transfected cells have shorter neurites in the presence of doxycycline compared to control cultures, which is statistically significant as determined by a two-tailed Student’s t-test (p<0.01). This reduction in neurite length is even more pronounced than the standard dominant negative Rit-35 mutant, suggesting that the SMN-induced Rit truncation product similarly acts as a dominant negative. Transfection of control culture with either Rit-38 or Rit-TM also resulted in significantly shorter neuritis, comparable to the decrease seen after SMN depletion, supporting the theory that Rit-TM may act as a dominant negative. In contrast, Rit-79 did not significantly increase neurite length in control cultures. Representative micrographs are shown in Fig 10e.

Rit1 has previously been shown to promote neuronal differentiation and survival by increasing p38 phosphorylation [50–52], activated p38 has been shown to increase SMN protein levels both *in vitro* and in animal models [53, 54], leading us to hypothesize that the recuse provided by constitutively active Rit-79 in the NSC-34 cell model might be a result of p38-induced increases in SMN protein even in the presence of doxycycline. To address this, NSC-34 cells were cultured with or without doxycycline, and transfected with either Rit-FL or Rit-79. Total cell lysates were interrogated by Western blot, and we were able to see that Rit-79 increased phosphorylation of p38 in both conditions, but particularly in SMN-depleted cells. In cultures transfected with Rit-79, SMN protein levels were increased and it appeared to completely blunt the doxycycline-induced knockdown leading to increased levels of SMN protein even in the presence of doxycycline compared to cells transfected with Rit-FL which did not restore neurite length (Fig 10F). Further study is required to determine the mechanism by which Rit1-tm reduces neurite outgrowth in these cells.

## Discussion

Defining a pathological mechanism by which SMN depletion results in neurodegeneration has remained elusive to date. No single molecular event, which when restored can fully compensate for the effect of SMN depletion on multiple genetic backgrounds, has been reported in SMA animal models. This is not surprising given the role of SMN in the assembly of a diverse range of RNP complexes. Whether SMN’s specific function in snRNP biogenesis and mRNA trafficking are mutually exclusive remains to be determined. To assess how SMN reduction affects gene expression profiling within a single cell model system of SMA, independent replicates of total mRNA were analyzed for RNA expression, isoform expression and mis-splicing events. SMN depletion resulted in multiple isoform changes indicative of the sensitivity of alternative splicing to changes in SMN protein levels. Examples of both elongated and truncated isoforms accompanying SMN depletion were evident, suggesting that in addition to exon skipping, exon retention events may also be observed in SMA conditions. A recent study used exon-exon micro-arrays to identify splicing errors in a severe mouse model and validated a subset of these changes in dividing neuroblastoma cells where SMN was depleted by siRNA [19]. Despite the fact that their experimental design in a cell-based system was similar to ours, we find very little overlap between our two datasets. This may partly be due to the fact that our cells are terminally differentiated to a more motor neuron-like phenotype whereas their N2a model was rapidly dividing and undifferentiated. Our interpretation of the absence of overlap in the two model systems is that while distinct SMN-dependent splicing changes are present among the test systems, the lack of a consistent constellation of errors implies that this cannot be the dominant cause of SMA pathology, although in our model system, the protein product resulting from aberrant Rit1 splicing appears to be a potential explanation for the reduction in neurite length seen after SMN depletion. Similarly, we found no overlap between our dataset and the report by Baumer and colleagues, which examined alternative splicing events in SMA mouse motor neurons at early, intermediate and late disease stages [55] despite the fact that RT-PCR analysis of spinal cords from symptomatic ‘Taiwanese” did show an increase in the alternatively spliced form of three exon-skipping and three intron-retention events that were validated in our NSC-34 cell model. There is a small degree of overlap with the aberrant splicing found by Zhang et al in late stage mice (Cenpt, Prc1, Ddx17, Krit1, Prmt1, Nup88, Pstk1, Sltm, Ptrh2, Cct4)[8], as well as a later study done in pre-symptomatic animals by laser-capture micro-dissection (Clstn1, Gpx8, Med7)[56]. In comparison, the small number of splicing changes identified after induction of SMA in adult mice shows no overlap with our dataset [17]. Together, these findings indicate that perhaps our cells most closely reflect SMN dependent splicing changes in the neonatal spinal cord rather than in undifferentiated cells or adult tissues. While this manuscript was undergoing revisions, a new study examined splice changes in a variety of tissues from the “Taiwanese” mouse model, which we used to validate our alternatively spliced products. They confirmed that *Srsf1* and *Rit1* were both alternatively spliced in SMA spinal cord as early as postnatal day 5 [57].

U12 introns have been proposed to be particularly susceptible sites for aberrant processing following SMN depletion. Using our approach, we were unable to identify many U12 introns retained in mRNA upon cross-reference with sequences within the U12 database (http://genome.crg.es/cgi-bin/u12db/u12db.cgi). Our data suggest that in NSC34 cells undergoing steady state differentiation with depleted SMN, minor-class introns do not display increased dependence on SMN. Similar findings were reported following RNA-seq analysis of laser capture motor neurons in SMA mouse spinal cord [56]. This partially supports recent data demonstrating embryonic lethality and locomotion in a *Drosophila* model of SMA that can be rescued by human SMN expression without a significant increase in snRNA levels [58]. However, without quantifying the required snRNA levels to restore these and other less significant splicing events, we cannot rule out that snRNP biogenesis is redundant in rescuing the motor neuronal phenotype in NSC34 cells.

To search for biological consequences of alternative splicing that could explain the shortened neurite phenotype in our NSC-34 cells model of SMA [21], we focused on intron retention events, as these often introduced a premature stop codon potentially encoding for a truncated protein product. Neuronal cells have proven able to translate intron-retained transcripts, even those which have been demonstrated targets of NMD. A splice site mutation in a GABA receptor subunit produces a retained intron, and the resultant protein product encodes a stable, truncated γ2 subunit that causes familial epilepsy [59]. A large number on intron-retaining transcripts have recently been reported in primary neuron cultures, indicating that neuronally cells may increase their transcriptional repertoire by making use of the alternatively spliced products in the cytoplasm rather than degrading them [60, 61].

The intron retention event with the largest difference in PSI between control and SMN-depleted samples was retention of a Vps33b 3’ intron following the normal stop codon, and is unlikely to produce any significant error in the protein product, although it could conceivably alter mRNA stability. The truncated murine Srsf1 protein appears to be unstable, as we could not detect it by Western blot. However, we were able to model the consequences of over-expressing the truncated product of the small G protein Rit1. Rit1 is alternatively spliced upon SMN depletion, creating a truncation mutant (Rit-TM) that reduced neurite length to a greater extent than even the commonly used S35N dominant negative mutant. This activity of Rit-TM is likely due to the loss of the G4 and G5 domains that are required for recognition of the guanine of GTP/GDP, as well as disruption of part of the effector protein binding site within the switch II domain. Only a few effector proteins have been discovered for Rit, and two of these play important roles in neurogenesis. Rit binds B-Raf and C-Raf but only activates the neuronal specific B-Raf, which plays a role in neurite outgrowth [50]. Par6C binding to Rit is rather unusual in that it is not GTP dependent, and loss of this interaction alters the fate of axon versus dendrite differentiation [62]. If Rit is unable to bind these effector proteins, the fate of neurite differentiation and outgrowth is likely to be compromised, which could play a role in the pathogenesis of SMA. Rit mutations in the switch II domain are associated with Noonan syndrome, which is partly characterized by cardiac defects [63]. Similar cardiac pathologies are found in the severe mouse model of SMA [64], and one could envision a scenario where severely low levels of SMN promote the accumulation of truncated Rit1, mimicking some Noonan syndrome pathologies.

The data presented here provide an interesting, albeit restricted snap shot of the complexity of SMN-dependent RNA processing. Even restricted to a clonal population of a single cell type, widespread abnormalities are evident that do not highlight any single cellular process or protein family, making therapeutic strategies for targeting compensatory mechanisms conceptually and technically challenging. It is likely that similar perturbation is present in additional cell types involved in the maintenance and maturation of the neuromuscular junction, providing an additional layer of complexity to the molecular pathogenesis of SMA, and highlighting the requirement to understand how non-neuronal phenotypes arise as a result of SMN depletion. Determining whether SMN function is spatiotemporally sensitive will be important in deciphering compensatory proteins and mechanism that can be manipulated in place of SMN restoration. The finding that over-expression of constitutively active Rit increased neurite length in SMN-depleted cultures demonstrates that correcting even a single SMN-dependent splice error has the power to restore aspects of normal neuronal architecture without increasing SMN protein levels. Whether correcting this single splice error is sufficient to rescue disease phenotype in a more complex model of SMA such as the zebrafish or mouse remains to be determined.

## Methods

### Cell culture

Murine neuroblastoma x spinal cord NSC-34 cells [20] were grown in DMEM (Gibco) supplemented to 10% with fetal calf serum (FCS) (Clontech) and 1% penicillin/streptomycin (PSt) at 37°C. Cells were grown to 80% confluence supplemented with or without 2 μg/ml doxycycline and split into at a density of 2.4x10^4^ cells/ml in DMEM:F12 (Gibco) supplemented with 1% FCS and 1% PSt to induce differentiation and promote neurite outgrowth in the presence of 2 μg/ml doxycycline or without. Cells were grown for 72 h until approximately 70% confluent with media changed after 48 h. Fibroblasts from a type II SMA patient (3813) and carrier parent (3814) were grown in DMEM (Gibco) supplemented to 15% with FCS (Sigma) and 1% PSt at 37°C to 50% confluence [65].

### High throughout sequencing

RNA samples were treated with Ribominus (Life Technologies) to reduce ribosomal RNA. RNA concentration was measured using the Agilent Bioanalyzer 2100, and 250–700 ngs of each individual RNA sample was fragmented with RNase III. Samples were processed through the Life Technologies SOLiD4 RNA Sequencing protocol; each individual sample was barcoded as part of the process. The barcoded libraries were pooled in equal amounts and processed through SOLiD4 EZ Bead preparation (Life Technologies) using 0.5 pM total in the final preparation. One full slide of 700-million template-beads was used for 50 base reads forward sequencing on Life Technologies SOLiD 4 Sequencer. The RNA-seq results are publically available at http://compbio.iupui.edu/group/6/pages/smn and have also been uploaded to the NCBI sequence read archive (SRP090323).

### Alternative splicing analysis and RBP enrichment analysis

Alternative splicing events including skipped exons (SE) and retained introns (RI) were detected using MISO with default parameters [25]. Reads from biological replicates were merged together to increase the power of splicing analysis. Differential splicing events with Bayes factor ≥ 10 were further used in motif enrichment analysis. The up-regulated and down-regulated events were analyzed separately. Each event was split to 7 regions: 150 bp up/down stream exons, whole skipped exons and 300 bp of their flanking introns. Sequences in each region were divided into bins based on GC content. All the combinations of 5-mer sequence patterns were searched in each region. The background frequency of 5-mer patterns were generated by 1^st^ order Markov Model [3]. The significance of each pattern was tested by Binomial test. Patterns with Benjamini-Hochberg corrected p-value ≤ 0.05 are considered as significantly enriched.

### Cloning

Full-length human Srsf1 (hSrsf1-FL) and truncation mutant (hSrsf1-TM) were cloned behind a HA-tag into pcDNA3. Full length contains all 4 coding exons, and TM includes exons 1–2 as well as the first 263 bp of intron 2. Protein encoded by TM stops 8 aa downstream of exon 2 due to a stop codon located in the intron. Mouse full length and truncation Srsf1 (mSrsf1-FL and mSrsf1-TM) were similarly cloned. mSrsf1-TM includes exons 1–2 followed by the first 264 bp of intron 2, so the encoded protein ends 47 aa downstream of exon 2. Full-length mouse Rit (mRit-FL) and truncation mutant (mRit-TM) were cloned behind a 4xFLAG tag into pCMV10. Full length encodes coding exons 2–6, while TM includes exons 2–4 plus the first 105 bp of intron 4 and encodes a protein that terminates 11 aa downstream of exon 4. All plasmids were confirmed by sequencing.

### Quantitative real-time PCR and end point PCR

Nucleic acid was isolated from NSC-34 using mirVana RNA isolation kit (Ambion) according to manufacturers instructions before DNase treatment using Turbo DNase kit (Ambion). Nucleic acid was isolated from NSC-34-4, NSC-34-4#56 and SMA patient fibroblasts for endpoint and qPCR analysis by lysing cell pellets in Trizol (Invitrogen) and following the manufacturers protocol up until resuspending the aqueous phase in ethanol, for further clean up and isolation RNA Clean & Concentrator -5 columns were used following manufacturers protocol (Zymo Research). 5 μg of RNA was used as input into cDNA synthesis from for all samples used in endpoint and qPCR using SuperScript III (Life Technologies) reverse transcriptase following manufacturers protocol. qPCR was performed using iQ SybrGreen Supermix (BioRad) and CFX96 real time system using 60°C annealing and extension temperature. Endpoint PCR was performed using GoTaq polymerase (Promega) with annealing temperatures of 55°C for differential isoforms, and 59.5°C for intronic transcript templates.

### Western blots

NSC34-4#56 cells were transfected with Lipofectamine 2000 at a 2:1 ratio with hSrsf1, mSrsf1, or mRit1 plasmids. After 24 hours, cells were lysed in 0.5% NP40/20mM Tris 8.0/150mM NaCl. Insoluble protein was pelleted, and 20–40 μg soluble fraction was run on 16% PAGE, transferred, blocked in milk/PBST, and blotted with FLAG M2 antibody at 1:5,000 dilution, HA-7 antibody at 1:40,000 dilution, or tubulin (Dm1a) at 1:50,000 dilution. Endogenous SMN was detected using MANSMA1 1:200 [66] and alpha-tubulin DM1A 1:10,000.

### Minigene assay

The SMN minigene assay to detect full length (+exon 7) or alternatively spliced (+ exon 7, Δ7) SMN was previously described [67] with one modification; the forward primer was replaced with pCI-Nhe-Xho-F to amplify only exogenous plasmid produced SMN.

### Immunofluorescence

NSC-34-4#56 cells were treated with 2 μg/mL Dox for 3 days in 10% FBS/DMEM. Glass coverslips (Fisher 12-545-100) were sterilized by UV irradiation and were treated with 1.5 μg/mL Poly-D-Lysine. SMN depleted NSC-34 or control cells were plated onto coverslips at 3x10^4^ cells/well and reverse transfected with Lipofectamine 2000 and Rit and pEGFP-C1. After 3 days, cells were washed with PBS and fixed in 4% paraformaldehyde/PBS and permeabilized in blocking solution (PBS, 5% normal goat serum, 0.1% Triton X-100) for 30 min. Alpha-tubulin antibody was diluted 1:2500 in blocking solution and incubated with cells for 2 hrs followed by Alexa-Fluor 594 1:1000. Slides were mounted using Prolong gold with DAPI, then visualized on a Nikon Microphot-SA microscope. Neurite lengths were measured using QCapture Pro 6.0 software. All lengths were normalized to GFP control transfected cells.

### Gene Ontology analysis

Gene Ontology analysis was performed using altered RNAs against the mouse mm9 genome.

### Animals and ethics statement

Transgenic mice and FVB/NJ breeders were obtained from Jackson Laboratories (stock # 005058 and 001800). The resultant progeny (Tg SMN2 2Hung^tg/O^; Smn1^TmHung/WT^) were intercrossed to produce “experimental” breeders (Tg SMN2 2Hung^O/O^; Smn1 ^TmHung/WT^). These were then mated with 005058 animals to produce litters which are 50% healthy (Tg SMN2 2Hung^tg/O^; Smn1^TmHung/WT-^) and 50% SMA (Tg SMN2 2Hung^tg/O^; Smn1^TmHung/ TmHung^). Animals were maintained in accordance with international guidelines with approval from the Indiana University School of Medicine Animal Care and Use Committee (IUSM IACUC) as described in protocol #10646. For spinal cord removal, pups were euthanized by decapitation at post-natal day 9 and total RNA was harvested as directed using the Trizol method detailed in the RNA micro kit (Invitrogen, # 12183016)

### Primers

mSrsf1-229-F TACGACTACGACGGCTACC

mSrsf1-447-R GTAACATACATCACCTGCC

mSrsf1-BamH1-F GATCGGATCCAGATGTCGGGAGGTGG

mSrsf1-Ale-Xho-R GTACCTCGAGCTTAAGTTATGTACGAGAGC

hSrsf1-227-F GCTATGATTACGATGGGTACC

hSrsf1-443-R CATACATCACCTGCTTCACGC

HindIII-HA-F GATCAAGCTTATGGCTTACCC

hSrsf1-Ale-Xho-R GTACCTCGAGCTTAAGTTATGTACGAGAGC

Hind-FLAG-mRit1-F GATCAAGCTTATGGTGGATTACAAGGATGACGACGATAAGTTAATTAAC ATGGAGTCCG

XbaBsu36-mRit1-R GTACTCTAGACCTGAGGTCAGGTGACCG

mRit-170F CTTATAAGATCCGGATCC

mRit-412R GGTCAGACTTGTTCC

Ex8Rev CTACAACACCCTTCTCACAG

pCI-Nhe-Xho-F GGCTAGCCTCG

mTrmt5 Ex2- F TATTTCCGAGCTGCACCAGA

mTrmt5 Ex2-R TCCTCTCCACACAGCACTTC

mFam64-a Ex2-3-F GAGAAGAAGGAGGTGACCCG

mFam64-a Ex2-3 R TCACGGATAAGGGAGACGGT

mCenpt i2-F AGGCAGGGTAGCCAAACAAA

mCenpt i2-R AACACGAGTCGATCTGCCAA

mPppc3cb-F CCGAGCAATTGGCAAGATGG

mPppc3cb-R CTCAGTGGTATGTGCGGTGT

mRit1 i2-F TCCGCATTGATGATGAACCT

mRit1 i2-R TCAGCTGCTTTAGGTCAGACT

mSrsF1 i2- F CCCGAGAGGCCGCTAT

mSrsF1 i2- R AGAAAACTGTATCCAATTCTGGC

mPik3c2a Ex2-F GAGATCGCCAAGTTGTCACC

mPik3c2a Ex2-R AGTAACTGGTAGTTGAAGCCCT

mPpp3cb qPCR F CCTAGTGGAGTGTTGGCTGG

mPpp3cb qPCR R CGGGGTGGCATTCTCTCATT

mTrmt5 qPCR F GCACCAGAGCATGAGAATCG

mTrmt5 qPCR R CGCTTTCCTAAGTCTCGGCA

mFam64a qPCR F ATCAAGGCTGAAGAGAGTGGTG

mFam64a qPCR R GACATCCTGGTGGCTTGGTG

mPik3c2a qPCR F GCGGGAGAAAAACATGGCTC

mPik3c2a wPCR R AATACCAGGACCTCACGCTG

mcenpt qPCR F GAACATGGCGGACCTCAG

mcenpt qPCR R AGTGCTCCGGCGTCTCAT

msrsf1 qPCR F ATAGCGTGGTGATCCTCTGC

msrsf1 qPCR R TGCCTACATCCGGGTTAAAG

hcenpt.exon2F GGAGAGCCCTGCTTGAAAC

hcenpt.exon3R CCAGCGCTCACTTACCAGTT

hsrsf1.ex 2F GCGGTCTGAAAACAGAGTGG

hsrsf1.ex 3R TGCCATCTCGGTAAACATCA

hrit.ex 4F GATGATGAGCCTGCCAATCT

hrit.ex 5R ACCCTTCTCCTGCCCTCATA

hPpp3cb Ex 12 F GACACTCAAGGGCCTGACTC

hPpp3cb Ex 14 R TCCTCGGGTGATCTGTCCAT

hPik3c2a ex 1 F GGATTACCTGGGCCTTCCAC

hPik3c2a ex 3 R CTGGGTTTGTGCGGTGATTG

hTrmt5 exon1 F: TCTACGAGCCTCTACCCCTG

hTrmt5 exon 3 R: CTGCTGAGGTGATTCCTGGAT

hFam64a exon 1 F: GAGTTTCAAATCGGCTGCGG

hFam64a exon 4 R: GCAGGCACTTCCTCAGTCA

mrit.I4.f CAGAACCGTCTGAGCAGTGT

mrit.I4.r CTGAAGCGCAGACTTCCAC

mcenpt.I2.f CCTCTACCCACTCACGTGCT

mcenpt.I2.r GACCCAAACTGCAGGTCCTA

msrsf.I2.f ACTTCGTGCGGGTTAAAATC

msrsf.I2.r TTGGGTAAATCACCACCACA

mPpp3cb Ex 12/14 F: CTGCAGTTTTGAAGAG

mPpp3cb Ex 14 R: CGGGGTGGCATTCTCTCATT

mFam64a ex 3 F: AGCTGTCTCAAAGGCTGGAC

mFam64a ex 4 R: ACGGATAAGGGAGACGGTCA

Pik3c2a Ex 1F: TTCTTGAAGAGAGATCGCCAA

Pik3c2a Ex 1/3 R: GGTCTTCAATCTGAGATACTTG

mTrmt5 Ex 1/3 F: GAGCATGAGGCCAAGTTATGG

mTrmt5 Ex 3 R: TTCCTCTCCACACAGCACTTC

mEF1a Int6F: CCGAGTGGAGACTGGTGTTC

mEF1a Int6R: AATCCAGAACAGGAGCGTAGC

## Supporting Information

S1 Fig Alternative Splicing after SMN depletion. A) End point RT-PCR shows that alternative splicing of *A2bp1* exon 19 is equal in all NSC-34 cell culture conditions and is unaffected by SMN depletion. 5s RNA is used as loading control. B) Quantitative RT-PCR shows total transcript levels after SMN depletion using primers in exons that are common to all splice variants. mRNA levels are shown after doxycycline-induced SMN depletion relative to controls by the ΔΔCT method using 5s RNA as the control gene. C) End-point RT-PCR using total RNA from either healthy parent fibroblasts (3814) or cells isolated from an SMA patient (3813). Only exon 13 of Ppp3cb shows is alternatively spliced in SMA patient fibroblasts compared to the parent. D) End-point RT-PCR using total RNA from either healthy parent fibroblasts (3814) or cells isolated from an SMA patient (3813). None of the validated SMN-dependent intron-retention events from the NSC-34 cell analysis were observed in patient fibroblasts.
(DOCX)

S2 Fig NSC-34 cell differentiation. A) Immunofluorescent micrograph of NSC-34 cells after 72 hours differentiation. Processes are mainly Map2 positive dendrites (green) and Map2 and tubulin (red) immunofluorescence overlaps completely. In the merged panel, nuclei are visualized with DAPI (blue). B) End-point RT-PCR expression of motor neuron markers in NSC-34 cell cultures after 72 hours of differentiation. C) Quantification of the percentage of cells producing a neurite at least twice the width of the soma after induction of differentiation. Error bars represent SEM from three independent cultures.
(DOCX)

## Abbreviations

SMN: survival motor neuron protein
SMA: spinal muscular atrophy
NSC: neuroblastoma x spinal cord
snRNP: small nuclear ribonucleic protein
RBP: RNA binding protein
NMJ: neuromuscular junction
rTta: reverse Tet-transactivator
