# Supplementary material for: Altered mRNA Splicing in SMN-Depleted Motor Neuron-Like Cells
Source: PLoS One. 2016 Oct 13;11(10):e0163954. doi: 10.1371/journal.pone.0163954 (PMC5063418; doi:10.1371/journal.pone.0163954)

**S1 Supporting Information**

**S1 Fig: Alternative Splicing after SMN depletion**

A) End point RT-PCR shows that alternative splicing of *A2bp1* exon 19 is equal in all NSC-34 cell culture conditions and is unaffected by SMN depletion. 5s RNA is used as loading control. B) Quantitative RT-PCR shows total transcript levels after SMN depletion using primers in exons that are common to all splice variants. mRNA levels are shown after doxycycline-induced SMN depletion relative to controls by the ∆∆CT method using 5s RNA as the control gene. C) End-point RT-PCR using total RNA from either healthy parent fibroblasts (3814) or cells isolated from an SMA patient (3813). Only exon 13 of Ppp3cb shows is alternatively spliced in SMA patient fibroblasts compared to the parent. D) End-point RT-PCR using total RNA from either healthy parent fibroblasts (3814) or cells isolated from an SMA patient (3813). None of the validated SMN-dependent intron-retention events from the NSC-34 cell analysis were observed in patient fibroblasts.


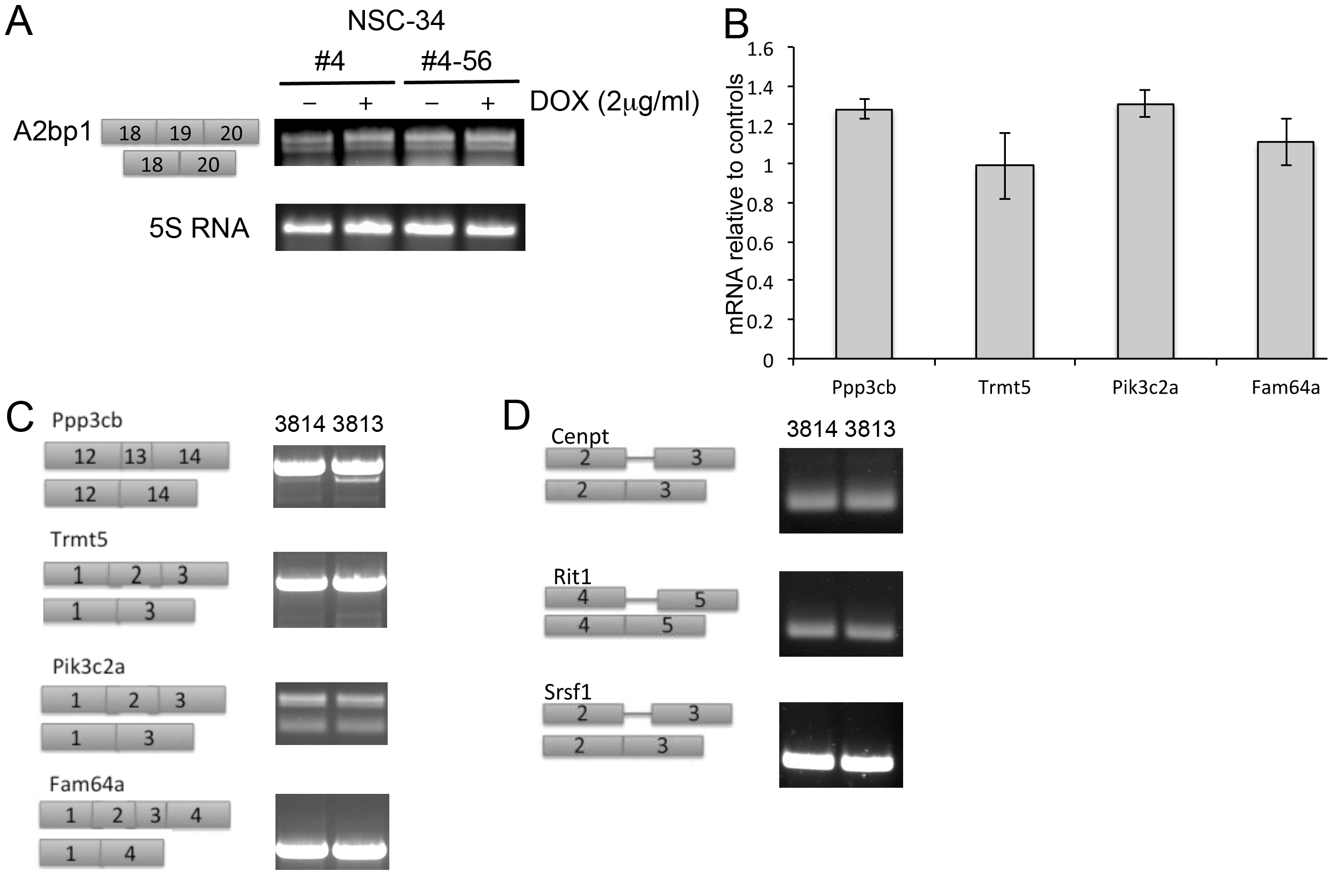

Supplement: S1 Fig — A) End point RT-PCR shows that alternative splicing of A2bp1 exon 19 is equal in all NSC-34 cell culture conditions and is unaffected by SMN depletion. 5s RNA is used as loading control. B) Quantitative RT-PCR shows total transcript levels after SMN depletion using primers in exons that are common to all splice variants. mRNA levels are shown after doxycycline-induced SMN depletion relative to controls by the ΔΔCT method using 5s RNA as the control gene. C) End-point RT-PCR using total RNA from either healthy parent fibroblasts (3814) or cells isolated from an SMA patient (3813). Only exon 13 of Ppp3cb shows is alternatively spliced in SMA patient fibroblasts compared to the parent. D) End-point RT-PCR using total RNA from either healthy parent fibroblasts (3814) or cells isolated from an SMA patient (3813). None of the validated SMN-dependent intron-retention events from the NSC-34 cell analysis were observed in patient fibroblasts. (DOCX) [file pone.0163954.s001.docx]
