# Supplementary material for: Altered mRNA Splicing in SMN-Depleted Motor Neuron-Like Cells
Source: PLoS One. 2016 Oct 13;11(10):e0163954. doi: 10.1371/journal.pone.0163954 (PMC5063418; doi:10.1371/journal.pone.0163954)

**S2 supporting information**

**S2 Fig: NSC-34 cell differentiation**

A) Immunofluorescent micrograph of NSC-34 cells after 72 hour differentiation. Processes are mainly Map2 positive dendrites (green) and Map2 and tubulin (red) immunofluorescence overlaps completely. In the merged panel, nuclei are visualized with DAPI (blue). B) End-point RT-PCR expression of motor neuron markers in NSC-34 cell cultures after 72 hours of differentiation. C) Quantification of the percentage of cells producing a neurite at least twice the width of the soma after induction of differentiation. Error bars represent SEM from three independent cultures.


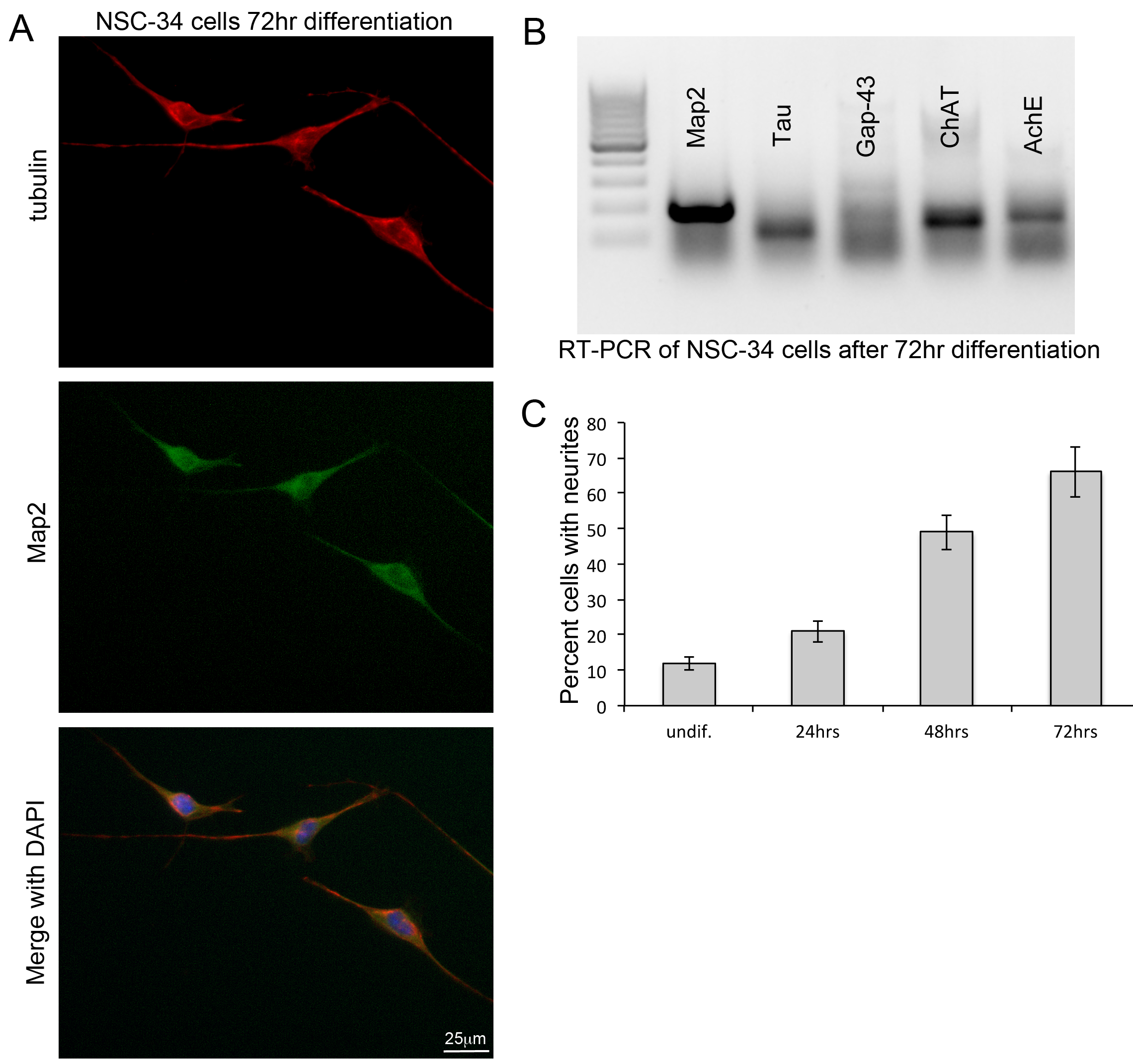

Supplement: S2 Fig — A) Immunofluorescent micrograph of NSC-34 cells after 72 hours differentiation. Processes are mainly Map2 positive dendrites (green) and Map2 and tubulin (red) immunofluorescence overlaps completely. In the merged panel, nuclei are visualized with DAPI (blue). B) End-point RT-PCR expression of motor neuron markers in NSC-34 cell cultures after 72 hours of differentiation. C) Quantification of the percentage of cells producing a neurite at least twice the width of the soma after induction of differentiation. Error bars represent SEM from three independent cultures. (DOCX) [file pone.0163954.s002.docx]
